# Microarray analysis of microRNA expression in the developing mammalian brain

**DOI:** 10.1186/gb-2004-5-9-r68

**Published:** 2004-08-31

**Authors:** Eric A Miska, Ezequiel Alvarez-Saavedra, Matthew Townsend, Akira Yoshii, Nenad Šestan, Pasko Rakic, Martha Constantine-Paton, H Robert Horvitz

**Affiliations:** 1 Howard Hughes Medical Institute, Department of Biology and McGovern Institute for Brain Research, Massachusetts Institute of Technology, Cambridge, Massachusetts 02139, USA; 2 Departments of Biology and Brain and Cognitive Sciences and McGovern Institute for Brain Research, Massachusetts Institute of Technology, Cambridge, Massachusetts 02139, USA; 3 Department of Neurobiology, Yale University School of Medicine, New Haven, Connecticut 06510, USA; 4 Center for Neurologic Diseases, Harvard Medical School, Boston, MA 02115, USA

## Abstract

**Background:**

MicroRNAs are a large new class of tiny regulatory RNAs found in nematodes, plants, insects and mammals. MicroRNAs are thought to act as post-transcriptional modulators of gene expression. In invertebrates microRNAs have been implicated as regulators of developmental timing, neuronal differentiation, cell proliferation, programmed cell death and fat metabolism. Little is known about the roles of microRNAs in mammals.

**Results:**

We isolated 18-26 nucleotide RNAs from developing rat and monkey brains. From the sequences of these RNAs and the sequences of the rat and human genomes we determined which of these small RNAs are likely to have derived from stem-loop precursors typical of microRNAs. Next, we developed a microarray technology suitable for detecting microRNAs and printed a microRNA microarray representing 138 mammalian microRNAs corresponding to the sequences of the microRNAs we cloned as well as to other known microRNAs. We used this microarray to determine the profile of microRNAs expressed in the developing mouse brain. We observed a temporal wave of expression of microRNAs, suggesting that microRNAs play important roles in the development of the mammalian brain.

**Conclusion:**

We describe a microarray technology that can be used to analyze the expression of microRNAs and of other small RNAs. MicroRNA microarrays offer a new tool that should facilitate studies of the biological roles of microRNAs. We used this method to determine the microRNA expression profile during mouse brain development and observed a temporal wave of gene expression of sequential classes of microRNAs.

## Abstract 2

A microarray technology suitable for analyzing the expression of microRNAs and of other small RNAs was used to determine the microRNA expression profile during mouse-brain development and observed a temporal wave of gene expression of sequential classes of microRNAs.

## Background

MicroRNAs constitute a large class of small regulatory RNAs [1]. Their mechanism of action and the scope of their biological roles are beginning to be understood. The first two microRNAs were discovered as the products of heterochronic genes that control developmental timing in *Caenorhabditis elegans *[2]. In heterochronic mutants, the timing of specific developmental events in several tissues is altered relative to the timing of events in other tissues. These defects result from temporal transformations in the fates of specific cells; that is, certain cells acquire fates normally expressed by cells at other developmental stages. The molecular characterization of the heterochronic gene *lin-4 *led to the surprising discovery that this gene encodes a 21-nucleotide non-coding RNA that regulates the translation of *lin-14 *mRNA through base-pairing with the *lin-14 *3' UTR [3,4]. A second heterochronic gene, *let-7*, encodes another small non-coding RNA that is conserved in flies and mammals [5].

Biochemical and bioinformatic approaches have identified many genes that encode microRNAs in *C. elegans*, plants, *Drosophila melanogaster *and mammals [6-18]. Like the *lin-4 *and *let-7 *genes, other microRNAs encode 21-25-nucleotide RNAs derived from longer transcripts that are predicted to form stem-loop structures. More than 200 microRNAs are encoded by the human genome [8,14].

The biological roles of microRNAs are poorly understood. In *C. elegans*, *lin-4 *and *let-7 *act in developmental timing, and the microRNA *lsy-6 *controls neuronal asymmetry [19]. In *Drosophila*, the microRNAs bantam and mir-14 act in the regulation of cell growth and cell death [20,21]. The mouse microRNA miR-181 has been implicated in the modulation of hematopoietic differentiation, and other mammalian microRNAs have been suggested to play roles in cancer [22,23].

Mature microRNAs are excised from a stem-loop precursor that itself can be transcribed as part of a longer primary RNA (pri-miRNA) [24]. The pri-miRNA appears to be processed by the RNAse Drosha in the nucleus, cleaving the RNA at the base of the stem-loop [25]. This cut defines one end of the microRNA. The precursor microRNA is then exported by Ran-GTP and Exportin-5 to the cytoplasm, where it is further processed by the RNAse Dicer [26,27]. Dicer recognizes the stem portion of the microRNA and cleaves both strands about 22 nucleotides from the base of the stem [25]. The two strands in the resulting double-stranded (ds) RNA are differentially stable, and the mature microRNA resides on the strand that is more stable [28,29]. Mature microRNAs can be found associated with the proteins eIF2C2 (an Argonaute-like protein), Gemin2 and Gemin3 and are thought to act in a protein-RNA complex with these and maybe other proteins [17,30].

The animal microRNAs studied so far act by reducing the levels of proteins from genes that encode mRNAs with sites partially complementary to microRNAs in their 3' UTRs [4,31]. The mechanism responsible is not understood in detail [32]. In contrast, although some plant microRNAs with partially complementary target sites also act by preventing translation, the majority studied so far cause the cleavage of target mRNAs at sites perfectly complementary to the microRNAs [33-38].

Determining spatial and temporal patterns of microRNA expression should yield insight into the biological functions of microRNAs. As the number of microRNAs identified has increased rapidly, the need for a method that allows for the parallel detection of microRNA expression has become apparent. Recent studies used a dot-blot technique to study 44 mouse microRNAs and northern blotting analysis to study 119 microRNAs from mouse and human organs [39,40].

In this study we cloned microRNAs from rat and monkey brains, developed a microRNA labeling method and used a microarray to monitor expression of microRNAs during mouse brain development. We determined the temporal expression pattern of 138 microRNAs in the mouse brain and found that the levels of 66 microRNAs changed significantly during development. We identified sets of genes with similar expression patterns, including genes that peaked in expression at different stages of development. More generally, the microRNA microarray we have developed can be used to determine the expression of all known microRNAs simultaneously under any set of experimental conditions or constraints.

## Results and discussion

### Identification of microRNAs from developing rat and monkey brains

**Figure 1:**
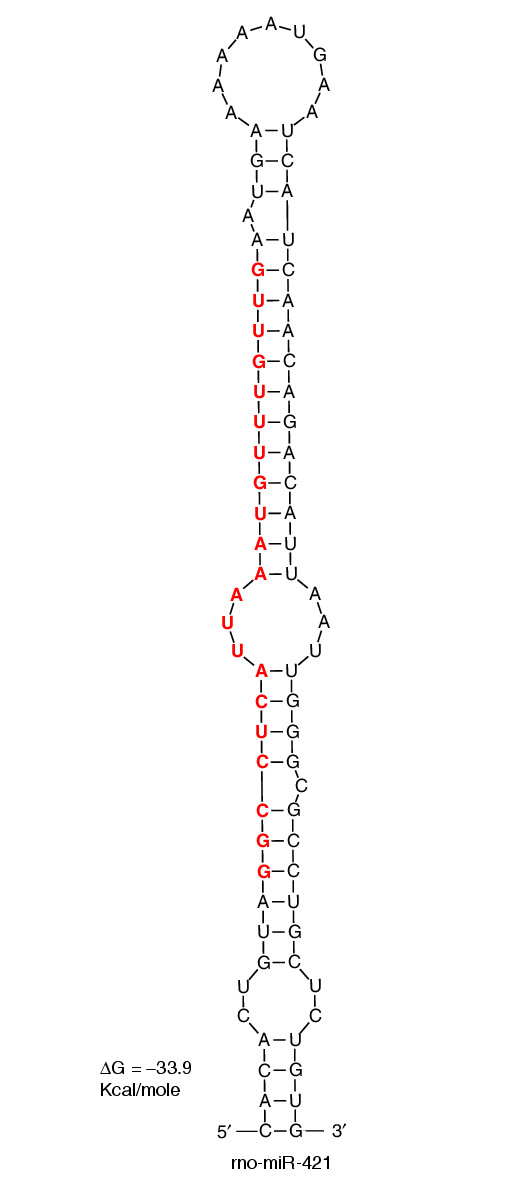
Predicted stem-loop structure of a novel mammalian microRNA, rno-miR-421. The stem-loop structure was predicted from sequences adjacent to rno-miR-421 in the rat genome. The cloned (mature) sequence is shown in red. The predicted secondary structure and the free energy calculation (ΔG, kcal/mole) were generated by the mfold software [52].

To analyze microRNAs expressed in the developing mammalian brain, we cloned small 18-26-nucleotide RNAs from the neocortex and hippocampus of a 12-day postnatal rat (*Rattus norvegicus*) and from the cerebral wall of a 114-day-old fetal rhesus monkey (*Macaca mulatta*) (Table 1). In both species, by these stages most neurons have been generated and have begun synaptogenesis [41,42]. We identified a total of 1,451 sequences, 413 of which correspond to microRNA sequences on the basis of their potential to generate stem-loop precursors as predicted from corresponding sequences in the rat and/or human genomes. In all cases but one, the microRNAs we identified corresponded to known microRNAs from other species and defined 68 unique microRNAs (Table 1 and Additional data file 1). One of these microRNAs is new: it differs in sequence from any microRNA previously described and is conserved in the mouse and human genomes. We named this new microRNA rno-miR-421 (Figure 1 and Additional data file 2). As observed in similar studies, in addition to microRNAs a number of candidate small RNAs that do not fulfill all criteria of a microRNA were cloned (Additional data file 3) [9,43]. Of the 52 rat microRNA sequences we cloned, 27 had previously been cloned from rat primary cortical neurons [11]. For 21 of the 52 microRNAs from rat and 14 of the 40 microRNAs from monkey we isolated only a single clone, indicating that our surveys are not saturated. By contrast, we isolated microRNA miR-124a 19 times from rat and 97 times from monkey. Mouse miR-124a as well as miR-128, miR-101 and miR-132 have been reported to be expressed specifically in brain [15]. We found that rat miR-138 also was expressed only in brain (Additional data file 4).

### MicroRNA microarrays for the study of temporal and spatial patterns of microRNA expression

Previous analyses of microRNA expression have relied on dot blots, northern blots and cloning strategies [8,11-14,18,39,40]. A highly scalable approach using a microarray would facilitate the analysis of microRNA expression patterns for a large number of samples and is feasible now that many mammalian microRNAs have been identified.

We arrayed 138 oligonucleotides complementary to microRNAs (probes) corresponding to the 68 mammalian microRNAs we isolated from rat and monkey brains, to 70 mammalian microRNAs isolated by others from a variety of mouse tissues and mammalian cell lines, and to predicted microRNAs. In addition, we included a set of control probes as well as 19 probes corresponding to presumptive small RNAs that we and others identified but that do not satisfy all the criteria for a microRNA (see below and Additional data file 5). Probes had a free amine group at the 5' terminus and were printed onto amine-binding glass slides and covalently linked to the glass surface. All probes were printed in quadruplicate (Additional data file 5).

We developed a method for preparing microRNA samples for microarray analysis. Several methods for mRNA sample labeling for microarray analysis have been described [44-47], but none is suitable for labeling RNAs as small as microRNAs. To fluorescently label small RNAs we adapted strategies for RNA ligation and reverse transcription PCR (RT-PCR) devised for microRNA cloning [12-14]. Briefly, 18-26-nucleotide RNAs were size-selected from total RNA using denaturing polyacrylamide gel electrophoresis (PAGE), oligonucleotide linkers were attached to the 5' and 3' ends of the small RNAs and the resulting ligation products were used as templates for an RT-PCR reaction with 10 cycles of amplification. The sense-strand PCR primer had a Cy3 fluorophore attached to its 5' end, thereby fluorescently labeling the sense strand of the PCR product. The PCR product was denatured and then hybridized to the microarray. As in microarray analysis, the labeled sample used for hybridization is referred to as the target. Significant biases in amplification, a problem when amplifying heterogeneously sized mRNAs, are less likely in the case of microRNAs because of their short uniform lengths. MicroRNA cloning frequencies obtained using a similar amplification strategy correlate well with expression levels as assayed by quantitative northern blots [7]. Because RNA is amplified before hybridization, relatively low amounts of starting material may be used with this method [8,11-14,18,39,40].

We optimized the conditions for hybridization to our microarray. The small sizes of microRNAs leave little opportunity for oligonucleotide (array probe) design to achieve homogeneous probe-target melting temperatures. Differences in melting temperatures are expected to result in greater nonspecific binding if hybridizations are performed at low temperatures (to allow the detection of probe-target pairs with low melting temperatures) and in less specific binding if hybridizations are performed at high temperatures (to specifically detect probe-target pairs with high melting temperatures). To assess this issue we included control probes with two internal mismatches on the microarray for a subset of the microRNA probes (Additional data file 5). We tested a range of hybridization temperatures, and, on the basis of the signal of microRNA probes versus control probes, we determined that a hybridization temperature of 50°C was a reasonable compromise between sensitivity and specificity (data not shown).

**Figure 2:**
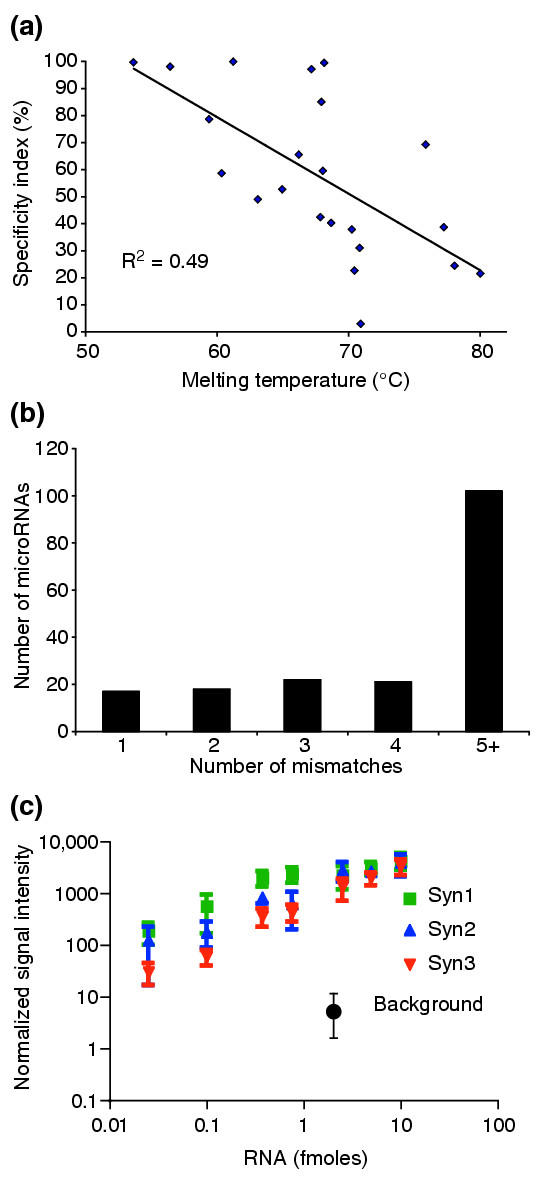
MicroRNA microarray specificity and quantification. **(a) **Specificity was assayed using a set of 23 microRNA and mismatched probe pairs (two mismatches). Average mean spot intensities from 10 independent hybridizations at 50°C were added to give a total signal for probes corresponding to a given microRNA as well as for probes with two mismatches to the microRNA. Mismatch probe design and sequences are described in Additional data file 6. A specificity index was calculated as 100 × (probe signal - mismatched probe signal)/probe signal. Melting temperatures for the microRNA probes were calculated using the nearest neighbors method [54]. The specificity index is plotted against the calculated melting temperature for each microRNA probe pair. Correlation of melting temperature and specificity index is significant (*p *= 0.004, Student's *t-*test). **(b) **Number of mismatches between microRNAs based on all known mouse microRNAs (the miRNA Registry 3.0 [53]). Each microRNA was aligned pairwise to every other microRNA and was assigned to the group (number of mismatches) corresponding to the least number of mismatches to another microRNA. **(c) **Quantification of microarray data using three synthetic RNAs: syn1, syn2 and syn3. Each data point is the average of two independent labelling/hybridization reactions. Probes for the three synthetic RNAs were printed in quintuplicate on the microarray. RNAs were used at 0.025, 0.1, 0.375, 0.75, 2.5, 5 and 10 fmoles. For comparison, the background signal of the array is shown. For more details, see Additional data file 5.

Even at 50°C, specificity as assayed by comparing microarray spot signal intensities from matched and mismatched probes varied among the microRNAs assayed. As expected, specificity at 50°C was negatively correlated with calculated melting temperatures (Figure 2a). In all cases the cumulative signal from 10 hybridizations for the mismatched probe was equal to or lower than that for the microRNA probe, but differences in the ratio of the matched to mismatched probe signal ranged widely (Figure 2a). Given these data, we do not expect the microRNA microarray to distinguish reliably between microRNAs that have only one or a few mismatches. This limitation is alleviated somewhat by the fact that for most microRNAs that have been identified the most closely related paralogs differ by five mismatches or more (Figure 2b). The signal from a mismatched control probe is likely to be caused by cross-hybridization with the microRNA for which it was designed, as other control probes corresponding to unrelated mRNA subsequences or synthetic probes that do not correspond to known microRNAs did not show signals above background (Additional data file 5). Microarray results for closely related microRNAs should be interpreted with caution, as differences in the apparent expression of a given microRNA could be dampened or exaggerated depending on the expression of the paralogs (Figure 2a).

To determine the detection range of the microarray, we synthesized three artificial RNAs with the characteristics of microRNAs. These RNAs were phosphorylated RNA oligonucleotides of 20-23 bases; their sequences were chosen at random and were without any significant sequence similarity to known mammalian microRNAs (see Additional data file 5 for details). We titrated these RNAs into total mouse RNA samples, labeled them and hybridized them to a microarray that in addition to microRNA probes included probes corresponding to these three RNAs, called syn1, syn2 and syn3. Figure 2c shows the correlation between the amount of the RNAs and the microarray signal intensities. For comparison, the background signal for the array is also shown. All three RNAs were reliably detected at levels as low as 0.1 fmoles. The dynamic range of the array was from 0.1 fmoles to at least 10 fmoles, or two orders of magnitude.

### Analysis of microRNA expression during mouse brain development

We isolated small RNAs from mice at five developmental stages: embryonic days 12.5 and 17.5 (E12.5 and E17.5), postnatal days 4 and 18 (P4 and P18) and 4-month-old adults. E12.5-E17.5 spans a period of major neuronal proliferation and migration in the mouse brain, in particular the birth and subsequent migration of most neurons in the ventricular zone epithelium [48]. Between postnatal days P4 and P18, major sensory inputs are established. For example, eye opening occurs around P13 and is thought to result in activity-dependent neuronal remodeling [49].

We purified and size-selected RNA from whole mouse brains. For each sample, the products of four independent RNA amplifications based on two independent RNA ligations were hybridized to the array. A detailed description of our analysis of the microarray data is presented in Additional data file 5. Of the 138 microRNAs and 19 small RNAs represented by the probe set, 116 (74%) were expressed robustly (more than 75-fold over the level of background controls) at least at one time point. Of these, 83 (71%) changed significantly during the period surveyed (analysis of variance, ANOVA, *p *< 0.001) and 66 (57%) changed more than twofold. Of the microRNAs we cloned from rat and monkey and for which probes against the corresponding mouse homologs were present on the microarray, we detected 97% robustly.

**Figure 3:**
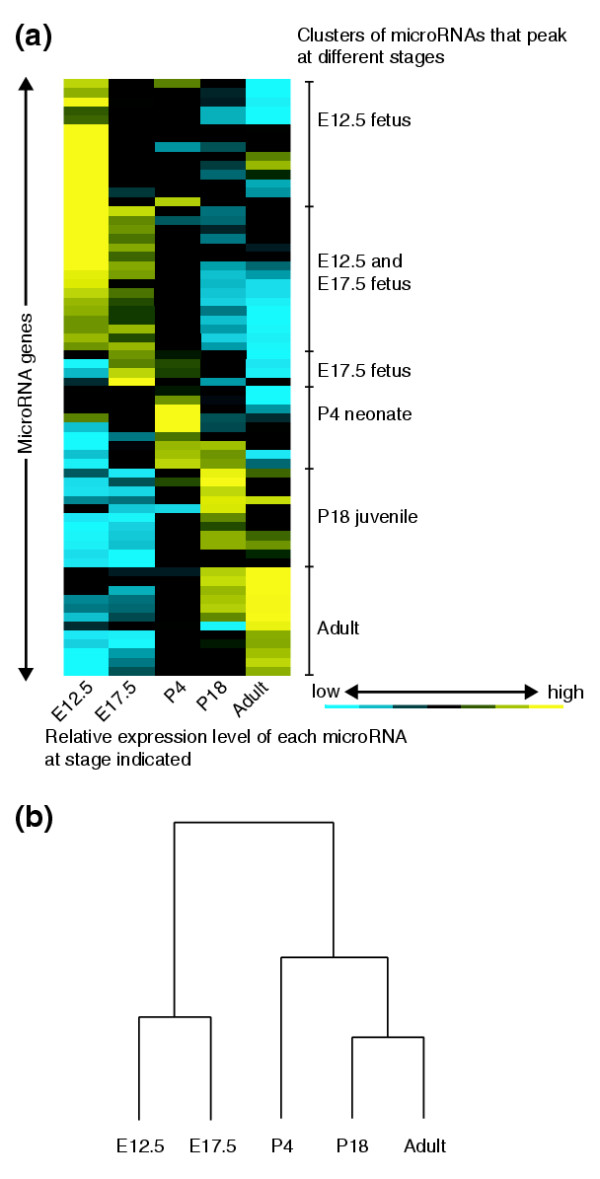
Profile of microRNA expression in the developing mouse brain. **(a) **Relative expression levels for the 66 microRNAs that changed significantly (ANOVA, *p *< 0.001) and more than twofold are shown in five columns corresponding to the five time points. Colors indicate relative signal intensities. The microRNA expression profile was sorted using a hierarchical clustering method, and major clusters are shown ordered according to the time that expression peaks. Gene names and a quantitative description of microRNA expression levels are presented in Additional data file 6. **(b) **Developmental time points were grouped using the same hierarchical clustering method and gene set as in (a).

**Figure 4:**
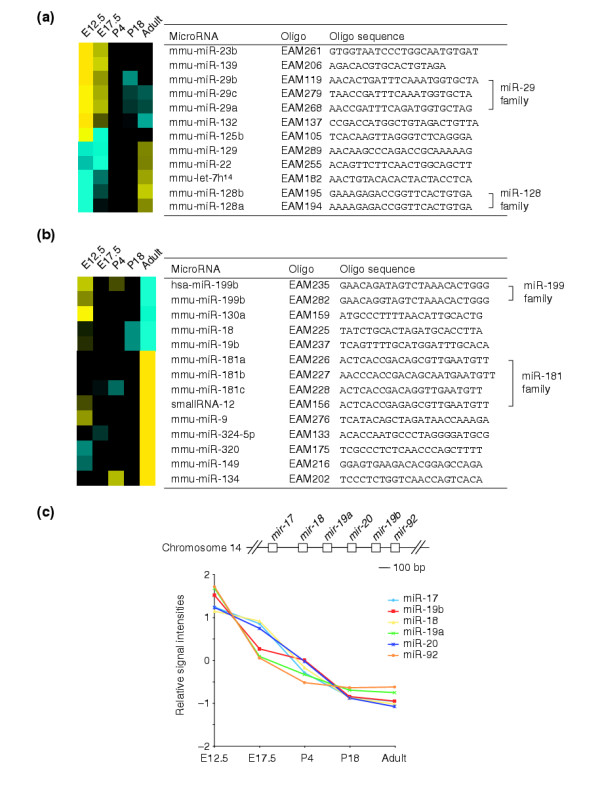
Examples of co-regulated microRNAs. **(a) **MicroRNAs with a sharp peak at the E12.5 stage. Methods were as described for Figure 3. Brackets indicate closely related sequences. **(b) **MicroRNAs with a single sharp peak at the adult stage. **(c) **Co-regulation of microRNAs derived from the *mir-17 *cluster from chromosome 14. To compare signal intensities, data were transformed to give a mean of 0 and a standard deviation of 1.

We grouped microRNAs that changed more than twofold in expression during the period analyzed using a hierarchical clustering algorithm (Figures 3a, 4) [50]. A group of microRNAs peaked at each of the developmental time points. The signal from 34 of the 66 probes that changed more than twofold peaked in the fetus (E12.5 and E17.5), suggesting roles in early development (Figure 4a). Nine and eleven microRNAs peaked during the neonate (P4) and juvenile (P18) stages, respectively. Twelve microRNAs had the highest signals at the adult stage (Figure 4b). These data indicate that murine brain development involves a wave of expression of sequential classes of microRNAs (Figure 3a).

We also grouped the developmental time points according to their microRNA expression pattern using hierarchical clustering. We found that samples from stages that are developmentally proximal had the most similar microRNA expression patterns (Figure 3b), indicating that a microRNA expression profile can be a marker of developmental stage. Examination of the temporal clusters revealed that probes with similar sequences showed correlated expression, as exemplified by miR-181a, miR-181b, miR-181c, smallRNA-12 (Figure 4a) and miR-29a, miR-29b and miR-29c (Figure 4b), respectively. Given our observation that the microRNA microarray can detect mismatched sequences, it is possible that this correlation among closely related family members is an artifact of hybridization.

We found that four of the 66 RNAs that changed more than twofold were small RNAs rather than microRNAs. The temporal regulation of these small RNAs indicates that they may play a role during development.

Several mouse microRNAs are clustered closely in the genome, suggesting that they might be expressed from a single precursor transcript or at least share promoter/enhancer elements. We searched all known microRNA clusters in the mouse genome to attempt to identify coordinately controlled clustered microRNAs. We sought clusters with the following features: first, the clustered microRNAs are not all members of the same family; second, the microRNAs have no or few paralogs; and third, the microRNAs are detected robustly on our microarray and their expression changes significantly during the timecourse studied. The *mir-17 *cluster on chromosome 14 fulfills all these criteria. Figure 4c shows that the expression of all six microRNAs in this cluster is indeed highly co-regulated.

### Validation of microarray results using northern blots

**Figure 5:**
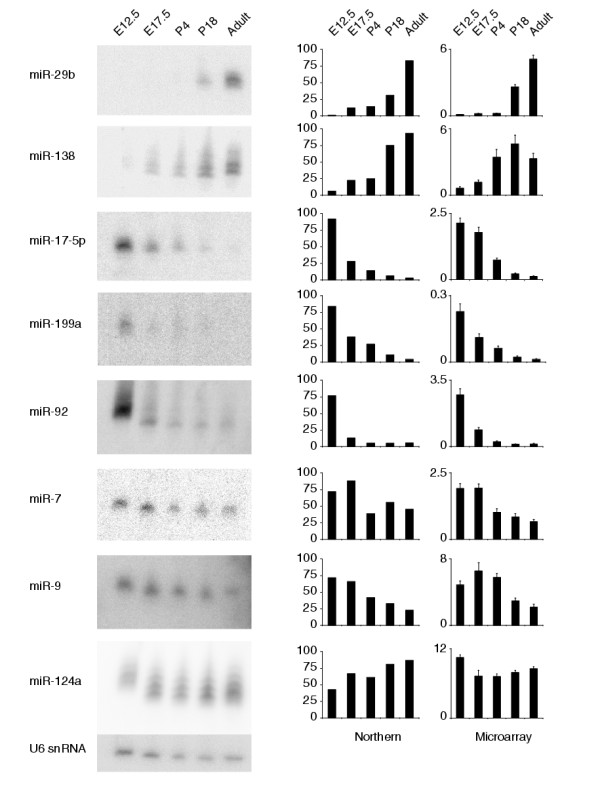
Comparison of microarray data with representative developmental northern blots of microRNAs. Northern blots were prepared and microarray analysis was done using the same starting material. For each microRNA, northern blots (left panel) and the microarray hybridization signals (right panel) are shown. Quantification of northern blots is also shown (middle panel). *Y*-axis for the microarray data refers to the averaged mean signal intensities (× 10^-3^), and error bars are standard errors of the mean. Northern blots were done using 20 μg of total RNA in each lane. Because northern blots were exposed for different lengths of time, the intensities of the signals on northern blots cannot be directly compared to those from the microarrays. A probe against U6 snRNA was hybridized to the same blots for comparison.

To validate our microarry results, we performed northern blots of eight microRNAs that were robustly expressed at least at one point during development according to our microarray data. The relative changes of microRNA expression assayed using microarray analysis and northern blots were consistent (Figure 5). For example, on a northern blot miR-29b was almost undetectable at the embryonic and P4 stages but appeared at P18 and was strongly expressed in the adult. The microarray data showed a similar pattern. In only a few cases did there seem to be discrepancies; for example, relative levels of expression of miR-138 at P4 compared to adult differed between the northern blots and the microarrays. As is the case for mRNAs, small differences may be seen between the methods and northern blot analysis is superior to microarrays for quantitative analysis [51]. Nonetheless, microarrays offer a high-throughput method that generally captures changes in microRNA expression.

## Conclusions

Here we describe the development of a microarray technology for profiling the expression of microRNAs and other small RNAs and apply this technology to the developing mammalian brain. Recently, Krichevsky *et al. *described the temporal expression of 44 microRNAs during mouse brain development [39]. Their study used a dot-blot array approach and direct labeling of microRNAs using radioactivity instead of a glass microarray and RT-PCR/fluorescent labeling, as we used in our study. Despite differences in sample selection as well as in the number of microRNAs analyzed, there is good agreement between the overlapping aspects of the two datasets. Our strategy has the potential to be highly scalable, allowing high-throughput analysis of samples with limiting starting material.

MicroRNA microarrays offer a new tool that should facilitate studies of the biological roles of microRNAs. We speculate that some of the developmentally regulated microRNAs we describe in this report play roles in the control of mammalian brain development, possibly by controlling developmental timing, by analogy to the roles of the *lin-4 *and *let-7 *microRNAs in *C. elegans*.

## Materials and methods

### MicroRNA cloning

We isolated RNAs and cloned microRNAs from *R. norvegicus *and *M. mulatta *using methods described previously [13], except that the samples were not dephosphorylated during the cloning procedure.

### Microarray printing and hybridization

Microarray probes were oligonucleotides (named EAM followed by a number) with sequences complementary to microRNAs. Each probe was modified with a free amino group linked to its 5' terminus through a 6-carbon spacer (IDT) and was printed onto amine-binding slides (CodeLink, Amersham Biosciences). Control probes contained two internal mismatches resulting in either C-to-G or T-to-A changes (Additional data file 6). Printing and hybridization were done using the protocols from the slide manufacturer with the following modifications: the oligonucleotide concentration for printing was 20 μM in 150 mM sodium phosphate pH 8.5, and hybridization was at 50°C for 6 h. Printing was done using a MicroGrid TAS II arrayer (BioRobotics) at 50% humidity.

### Sample and probe preparation

Whole brains from three to eight C57BL/6 mice were pooled. Starting with 250 μg of total RNA for each time point, 18-26-nucleotide RNA was purified on denaturing PAGE gels. The samples were divided, and the following cloning steps were done independently twice for each time point. 3' and 5' adaptor oligonucleotides were ligated to 18-26-nucleotide RNA followed by reverse transcription, essentially as described for microRNA cloning [13]. Briefly, a RNA-DNA hybrid 5'-pUUUaaccgcgaattccagt-idT-3' (Dharmacon: X, RNA; x, DNA; p, phosphate; idT, inverted [3'-3' bond] deoxythymidine) was ligated to the 3' end and 5'-acggaattcctcactAAA-3' (Dharmacon) was ligated to the 5' end. The ligation products were divided into two aliquots, and the following steps were done independently twice for each time point. Ligation products were reverse transcribed and amplified by 10 rounds of PCR (40 sec at 94°C, 30 sec at 50°C, 30 sec at 72°C). For PCR, the oligonucleotides used were: oligo1 5'-Cy3-ACGGAATTCCTCACTAAA-3' and oligo2 5'-TACTGGAATTCGCGGTTAA-3'. The PCR product was precipitated, washed and resuspended in hybridization buffer (5× SSC, 0.1% SDS, 0.1 mg/ml sheared denatured salmon sperm DNA).

### Data acquisition and analysis

Microarray slides were scanned using an arrayWoRx biochip reader (Applied Precision), and primary data were analyzed using the Digital Genome System suite (MolecularWare) and Spotfire DecisionSite (Spotfire). Cluster analysis was performed using the CLUSTER/TreeView software [50]. For details concerning microarray data analysis see Additional data file 5. The predicted stem-loop RNA structures were generated using the mfold (version 3.1) software [52].

### Northern blots

Northern blots were performed as described [14]. Twenty micrograms of total RNA were loaded per lane. A probe for the mouse U6 snRNA (5'-TGTGCTGCCGAAGCGAGCAC-3') was used as loading control. The probes for the northern blots had the same sequences as the corresponding EAM oligonucleotides printed on the microarray (see Additional data file 6). The blots were stripped by boiling for 5 min in distilled water and reprobed up to four times.

The probes used were: EAM119 (miR-29b), EAM125 (miR-138), EAM224 (miR-17-5p), EAM234 (miR-199a), EAM131 (miR-92), EAM109 (miR-7), EAM150 (miR-9) and EAM103 (miR-124a).

## Additional data files

The following additional data files are available with the online version of this article. A file (Additional data file 1) with details of rat microRNA precursors: using the assembly of the rat genome [55] we identified candidate genomic locations for all of our rat microRNAs that have orthologs in the mouse but that have not been described previously for the rat. An alignment of the top BLAST hit of each mouse microRNA precursor sequence (The miRNA Registry, Release 3.2) against the rat genome sequence (public release draft genome assembly, version 3.1) is shown in this file. In addition, predicted precursor secondary structures are shown for each rat microRNA gene for which the precursor sequence differs from that of the corresponding mouse precursor. We used the mfold algorithm to make secondary structure predictions [52].

A file (Additional data file 2) containing precursor sequences and secondary structure predictions for the novel microRNA miR-421. An alignment of the predicted precursor sequences from human and mouse of the novel microRNA miR-421, which we identified from rat, is shown. The cloned sequence (corresponding to the mature microRNA) is shown in bold. The single mismatch is indicated by an asterisk (*). The accession number for each sequence is given. The predicted secondary structures for the corresponding genes from mouse and human are also shown. The human and mouse genomic sequences for candidate miR-421 precursors are identical. The sequence of the mature microRNA is colored in red. The residue in the rat genomic sequence that is different from the mouse and human genomic sequences is indicated. We used the mfold algorithm to make secondary structure predictions [52]. We have not been able to detect miR-421 in mouse brain using northern blots.

A file (Additional data file 3) with details of other small RNAs cloned from rat and monkey brains. We cloned 13 small RNAs that do not satisfy all criteria to be considered microRNAs. One, small RNA-1 from monkey, is present in the mouse genome and has a predicted stem-loop precursor sequence characteristic of microRNAs. However, the predicted stem-loop ends on the final base of the microRNA, which is not typical for microRNAs. To be conservative, we refer to this RNA as a small non-coding RNA. A northern blot for smallRNA-1 revealed a high molecular weight band that may represent a precursor RNA (data not shown). Since there is no perfect match to smallRNA-1 in the current release of the human sequence, a presumptive precursor based on mouse genomic sequence is shown. The cloned sequence is highlighted in red. The other small RNAs are not predicted to form stem-loop structures.

A file (Additional data file 4) showing rno-miR-138 brain specific expression. A northern blot shows that rno-miR-138 expression was restricted to brain. The probe was identical to EAM125. Total RNA isolated from various adult rat tissues (Ambion) was size-separated on denaturing PAGE (12 μg per lane), transferred to a nylon membrane and used for hybridization. Equal loading was verified using a probe for U6 snRNA (data not shown).

A file (Additional data file 5) with details of microarray design and data analysis.

And a file (Additional data file 6) with a summary of the microRNA microarray data. Oligonucleotide sequences correspond to probes on the array. MicroRNA names were obtained from the miRNA registry (Release 3.2) or if not available these were obtained from NCBI. Probes named smallRNA-1 through -13 correspond to unique small RNAs that we cloned but that did not correspond to known microRNAs and did not have perfect matches in the current release of the rat genome sequence. Column A indicates whether the probe is complementary to a microRNA or to one of the small RNAs we cloned ("-" = no, "+" = yes). Column B indicates whether the probe is complementary to a mouse microRNA. Oligonucleotides with a "-" in column A were either controls or sequences that were submitted to public databases as microRNAs and later found not to encode microRNAs. In a few cases we printed probes that represented the same microRNA twice, but we analyzed the data from only one of these probes. The probes we did not analyze had no labels in columns A, B, and C. Melting temperatures were calculated using the nearest neighbors method [53]. Data for the five time points of mouse brain development (E12.5, E17.5, P4, P18 and adult) are shown. Microarray data were derived as described (Additional data file 5). Briefly, data correspond to mean spot intensities averaged over quadruplicates (on each array) and four independent hybridizations. SEM refers to the standard error of the mean. microRNAs labeled with ^% ^are not in the current release of the miRNA registry but are deposited in NCBI and described elsewhere [8,15,18].

An Excel file (Additional data file 7) with the primary microarray data corresponding to the E12.5 time point; an Excel file (Additional data file 8) with the primary microarray data corresponding to the E17.5 time point; an Excel file (Additional data file 9) with the primary microarray data corresponding to the P4 time point; an Excel file (Additional data file 10) with the primary microarray data corresponding to the P18 time point; an Excel file (Additional data file 11) with the primary microarray data corresponding to the Adult time point. All data in Additional files 7-11 were exported from the Digital Genome System suite (MolecularWare). The primary microarray data will also be submitted to the Gene Expression Omnibus (GEO) database [56].

## Supplementary Material

Additional data file 1 A file with details of rat microRNA precursors Click here for additional data file

Additional data file 2 A file containing precursor sequences and secondary structure predictions for the novel microRNA miR-421 Click here for additional data file

Additional data file 3 A file with details of other small RNAs cloned from rat and monkey brains Click here for additional data file

Additional data file 4 A file showing rno-miR-138 brain specific expression Click here for additional data file

Additional data file 5 A file with details of microarray design and data analysis Click here for additional data file

Additional data file 6 A file with a summary of the microRNA microarray data Click here for additional data file

Additional data file 7 An Excel file with the primary microarray data corresponding to the E12.5 time point Click here for additional data file

Additional data file 8 An Excel file with the primary microarray data corresponding to the E17.5 time point Click here for additional data file

Additional data file 9 an Excel file with the primary microarray data corresponding to the P4 time point Click here for additional data file

Additional data file 10 An Excel file with the primary microarray data corresponding to the P18 time point Click here for additional data file

Additional data file 11 An Excel file with the primary microarray data corresponding to the Adult time point Click here for additional data file

## Figures and Tables

**Table 1:** Identity, frequency and size range of microRNAs cloned from the cortex and hippocampus of 12-day postnatal *R. norvegicus *and the cortex of a 114-day old *M. mulatta *fetus

*Rattus norvegicus *microRNAs			*Macaca mulatta *microRNAs		
	
Name	Number of times cloned	Size range	Name	Number of times cloned	Size range
rno-miR-421	2	21			
rno-let-7a	3	22	mml-let-7a	15	21
			mml-let-7a or c	1	18
rno-let-7b	1	23	mml-let-7b	20	22-23
rno-let-7c	10	22	mml-let-7c	9	21-22
rno-let-7d	1	22	mml-let-7d	1	22
			mml-let-7e	3	20-22
			mml-let-7f	3	22
			mml-let-7g	2	22
rno-let-7i	1	22	mml-let-7i	2	22
rno-miR-7	5	21			
			mml-miR-7-1	1	22
rno-miR-9	2	23	mml-miR-9	9	21-23
rno-miR-16	2	22	mml-miR-16	2	22
rno-miR-17-5p	3	23	mml-miR-17-5p	2	22-23
rno-miR-24	6	21-22			
			mml-miR-26a	3	21-22
rno-miR-26b	1	22			
rno-miR-28	1	22			
rno-miR-29a	4	22			
rno-miR-29b	7	22-23			
rno-miR-29c	2	20,22			
rno-miR-30b	1	22	mml-miR-30b	2	22
rno-miR-30c	3	23-24	mml-miR-30c	1	21
			mml-miR-33	2	20
rno-miR-92	2	22	mml-miR-92		
rno-miR-93	1	23			
rno-miR-99a	1	21	mml-miR-99a	4	20-22
rno-miR-99b	2	21,22	mml-miR-99b	2	22
			mml-mir-100	1	22
rno-miR-101b	1	22			
rno-miR-103	3	23	mml-miR-103	2	22-23
			mml-miR-103 or 107	1	21
rno-miR-124a	19	19-22	mml-miR-124a	97	18-23
rno-miR-125a	2	22,24	mml-miR-125a	4	22-23
rno-miR-125b	12	21-22	mml-miR-125b	17	20-22
			mml-miR-126	1	21
			mml-miR-126*	1	22
rno-miR-127	1	20			
rno-miR-128a	3	21-22	mml-miR-128a	9	22
rno-miR-128a or b	2	21	mml-miR-128a or b	17	18-21
rno-miR-128b	1	21	mml-miR-128b	8	22
rno-miR-129	2	21-22	mml-miR-129-2	1	22
rno-miR-130a	1	22			
rno-miR-132	6	22			
rno-miR-136	2	23	mml-miR-136	1	23
			mml-miR-137	1	23
rno-miR-138	5	23-24			
rno-miR-139	1	23			
			mml-miR-140	1	22
rno-miR-140*	1	22			
rno-miR-142-3p	1	23			
rno-miR-145	1	23	mml-miR-145	2	22
rno-miR-146	2	23			
			mml-miR-149	2	23
rno-miR-150	4	22-23			
rno-miR-154	1	22			
			mml-miR-181a or 213	4	20-25
			mml-miR-181b	1	24
			mml-miR-181c	1	21
rno-miR-185	2	22-23	mml-miR-185	1	23
rno-miR-191	3	23-24			
			mml-miR-195	1	22
rno-miR-213	1	22			
			mml-miR-221	3	22-23
rno-miR-300	1	21			
rno-miR-323	1	22			
rno-miR-324	4	23			
rno-miR-325	1	22			
rno-miR-338	5	23			
rno-miR-342	1	25			
rno-miR-345	1	22			
Total	152		Total	261	

The rat (rno) and monkey (mml) microRNA names are indicated. Two microRNA names are assigned to the same clone when the cloned sequence is too short to distinguish between the microRNAs. mml-miR-7 and mml-miR-129 are encoded by three and two distinct genomic loci, respectively, although the sequences immediately adjacent to these microRNA sequences differ. The sequences we cloned for mml-miR-7-1 and mml-miR-129-2 were one base longer than that shared by the microRNAs, allowing us to determine the loci from where they originated, as indicated by -1 and -2. Notation follows the miRNA registry guidelines [53].
