# Supplementary material for: Microarray analysis of microRNA expression in the developing mammalian brain
Source: Genome Biol. 2004 Aug 31;5(9):R68. doi: 10.1186/gb-2004-5-9-r68 (PMC522875; doi:10.1186/gb-2004-5-9-r68)
Supplement: Additional data file 1 — A file with details of rat microRNA precursors [file gb-2004-5-9-r68-s1.pdf]

mmu-let-7a-1 *Mus musculus* let-7a-1 precursor RNA

```
>gi|34874105|ref|NW_047490.1|Rn17_2012 Rattus norvegicus chromosome 17 WGS
               supercontig

Mouse: 1       ttcactgtgggatgaggtagtaggttgtatagttttagggtcacacccaccactgggaga 60
               ||||||||||||||||||||||||||||||||||||||||||||||||||||||||||||
Rat:   3523419 ttcactgtgggatgaggtagtaggttgtatagttttagggtcacacccaccactgggaga 3523478

Mouse: 61      taactatacaatctactgtctttcctaaggtgat 94
               ||||||||||||||||||||||||||||||||||
Rat:   3523479 taactatacaatctactgtctttcctaaggtgat 3523512
```

mmu-let-7a-2 *Mus musculus* let-7a-2 precursor RNA

```
>gi|34865068|ref|NW_047799.1|Rn8_2323 Rattus norvegicus chromosome 8 WGS
                supercontig

Mouse: 3        gcatgttcccaggttgaggtagtaggttgtatagtttagagttacatcaagggagataac 62
                ||||| ||||||| |||||||||||||||||||||||||||||||| |||||||||||||
Rat:   14144995 gcatgctcccaggctgaggtagtaggttgtatagtttagagttacaacaagggagataac 14145054

Mouse: 63       tgtacagcctcctagctttccttgggacttgcac 96
                ||||||||||||||||||||||||||||||||||
Rat:   14145055 tgtacagcctcctagctttccttgggacttgcac 14145088
```

mmu-let-7b *Mus musculus* let-7b precursor RNA

```
>gi|34934010|ref|NW_047783.1|Rn7_2307 Rattus norvegicus chromosome 7 WGS
               supercontig

Mouse: 4       gggtgaggtagtaggttgtgtggtttcagggcagtgatgttgcccctccgaagataacta 63
               |||||||||||||||||||||||||||||||||||||||| |||||||||||||||||||
Rat:   1610759 gggtgaggtagtaggttgtgtggtttcagggcagtgatgtcgcccctccgaagataacta 1610818

Mouse: 64      tacaacctactgccttccctga 85
               ||||||||||||||||||||||
Rat:   1610819 tacaacctactgccttccctga 1610840
```

mmu-let-7c-1 *Mus musculus* let-7c-1 precursor RNA

```
>gi|34933964|ref|NW_047354.1|Rn11_1874 Rattus norvegicus chromosome 11 WGS
                supercontig

Mouse: 1        tgtgtgcatccgggttgaggtagtaggttgtatggtttagagttacaccctgggagttaa 60
                ||||||||||||||||||||||||||||||||||||||||||||||||||||||||||||
Rat:   16398264 tgtgtgcatccgggttgaggtagtaggttgtatggtttagagttacaccctgggagttaa 16398323

Mouse: 61       ctgtacaaccttctagctttccttggagcacact 94
                ||||||||||||||||||||||||||||||||||
Rat:   16398324 ctgtacaaccttctagctttccttggagcacact 16398357
```

mmu-let-7c-2 *Mus musculus* let-7c-2 precursor RNA

```
>gi|34934010|ref|NW_047783.1|Rn7_2307 Rattus norvegicus chromosome 7 WGS
               supercontig

Mouse: 1       acggcctttggggtgaggtagtaggttgtatggttttgggctctgccccgctctgcggta 60
               ||||||||||||||||||||||||||||||||||||||||||||||||||||||||||||
Rat:   1610341 acggcctttggggtgaggtagtaggttgtatggttttgggctctgccccgctctgcggta 1610400

Mouse: 61      actatacaatctactgtctttcctgaagtggccgc 95
               |||||||||||||||||||||||||||||||||||
Rat:   1610401 actatacaatctactgtctttcctgaagtggccgc 1610435
```

mmu-let-7e *Mus musculus* let-7e precursor RNA

```
>gi|34854887|ref|NW_047555.1|Rn1_2077 Rattus norvegicus chromosome 1 WGS
               supercontig

Mouse: 1       cgcgcccccgggctgaggtaggaggttgtatagttgaggaagacacccgaggagatcac 60
               |||||||||||||||||||||||||||||||||||||||||||||||||||||||||||
Rat:   1805450 cgcgcccccgggctgaggtaggaggttgtatagttgaggaagacacccgaggagatcac 1805509

Mouse: 61      tatacggcctcctagctttccccaggctgcgcc 93
               |||||||||||||||||||||||||||||||||
```

```
Rat:   1805510 tatacggcctcctagctttccccaggctgcgcc 1805542
```

hsa-let-7i Homo sapiens let-7i precursor RNA

>gi|34865737|ref|NW_047776.1|Rn7_2300 *Rattus norvegicus* chromosome 7 WGS
supercontig

```
Mouse: 1       ctggctgaggtagtagtttgtgctgttggtcgggttgtgacattgcccgctgtggagata 60
               ||||||||||||||||||||||||||||||||||||||||||||||||||||||||||||
Rat:   3609065 ctggctgaggtagtagtttgtgctgttggtcgggttgtgacattgcccgctgtggagata 3609006

Mouse: 61      actgcgcaagctactgccttgcta 84
               ||||||||||||||||||||||||
Rat:   3609005 actgcgcaagctactgccttgcta 3608982
```

mmu-mir-7-1 *Mus musculus* miR-7-1 precursor RNA

>gi|34873863|ref|NW_047487.1|Rn17_2009 *Rattus norvegicus* chromosome 17 WGS
supercontig

```
Mouse: 1       ttggatgttggcctagttctgtgtggaagactagtgattttgttgtttttagataactaa 60
               ||||||||||||||||||||||||||||||||||||||||||||||||||||||||||||
Rat:   6715460 ttggatgttggcctagttctgtgtggaagactagtgattttgttgtttttagataactaa 6715519

Mouse: 61      aacgacaacaaatcacagtctgccatatggcacaggccacctctacag 108
                |||||||||||||||||||||||||||||||||||||||||||||||
Rat:   6715520 gacgacaacaaatcacagtctgccatatggcacaggccacctctacag 6715567
```

mmu-mir-7-2 *Mus musculus* miR-7-2 precursor RNA

>gi|34857743|ref|NW_047560.1|Rn1_2082 *Rattus norvegicus* chromosome 1 WGS
supercontig

```
Mouse: 8        ccagccccgtttggaagactagtgattttgttgttgtgtctctgtatccaacaacaagtc 67
                ||||||| || |||||||||||||||||||||||||||||| |||  |||||||||||||
Rat:   14956730 ccagccctgtctggaagactagtgattttgttgttgtgtctgtgt--ccaacaacaagtc 14956787

Mouse: 68       ccagtctgccacatggtgctggtca 92
                |||||||||||||||||| ||||||
Rat:   14956788 ccagtctgccacatggtgttggtca 1495681Q
```

mmu-mir-9-1 *Mus musculus* miR-9-1 precursor RNA

>gi|34858271|ref|NW_047626.1|Rn2_2148 *Rattus norvegicus* chromosome 2 WGS
supercontig

```
Mouse: 1       cggggttggttgttatctttggttatctagctgtatgagtggtgtggagtcttcataaag 60
               ||||||||||||||||||||||||||||||||||||||||||||||||||||||||||||
Rat:   6714042 cggggttggttgttatctttggttatctagctgtatgagtggtgtggagtcttcataaag 6714101

Mouse: 61      ctagataaccgaaagtaaaaataacccca 89
               |||||||||||||||||||||||||||||
Rat:   6714102 ctagataaccgaaagtaaaaataacccca 6714130
```

mmu-mir-9-3 *Mus musculus* miR-9-3 precursor RNA

>gi|34857743|ref|NW_047560.1|Rn1_2082 *Rattus norvegicus* chromosome 1 WGS
supercontig

```
Mouse: 1        ggaggcccgtttctctctttggttatctagctgtatgagtgccacagagccgtcataaag 60
                ||||||||||||||||||||||||||||||||||||||||||||||||||||||||||||
Rat:   15603655 ggaggcccgtttctctctttggttatctagctgtatgagtgccacagagccgtcataaag 15603714

Mouse: 61       ctagataaccgaaagtagaaatgactct 88
                ||||||||||||||||||||||||||||
Rat:   15603715 ctagataaccgaaagtagaaatgactct 15603742
```

mmu-mir-9-2 *Mus musculus* miR-9-2 precursor RNA

>gi|34853324|ref|NW_047616.1|Rn2_2138 *Rattus norvegicus* chromosome 2 WGS
supercontig

```
Mouse: 1       gttgttatctttggttatctagctgtatgagtgtattggtcttcataaagctagataacc 60
               ||||||||||||||||||||||||||||||||||||||||||||||||||||||||||||
Rat:   6903262 gttgttatctttggttatctagctgtatgagtgtattggtcttcataaagctagataacc 6903203
```

```
Mouse: 61      gaaagtaaaaac 72
               ||||||||||||
Rat:   6903202 gaaagtaaaaac 6903191
```

mmu-mir-16-2 ***Mus musculus*** **miR-16-2 precursor RNA**

>gi|34857808|ref|NW_047625.1|Rn2_2147 *Rattus norvegicus* chromosome 2 WGS supercontig

```
Mouse: 5        cttgttccactctagcagcacgtaaatattggcgtagtgaaataaatattaaacaccaat 64
                |||||||| |||||||||||||||||||||||||||||||||||||||||||||||||||
Rat:   45354558 cttgttccgctctagcagcacgtaaatattggcgtagtgaaataaatattaaacaccaat 45354617

Mouse: 65       attattgtgctgctttagtgtgacagggata 95
                |||||||||||||||||||||||||||||||
Rat:   45354618 attattgtgctgctttagtgtgacagggata 45354648
```

mmu-mir-24-1 ***Mus musculus*** **miR-24-1 precursor RNA**

>gi|34873863|ref|NW_047487.1|Rn17_2009 *Rattus norvegicus* chromosome 17 WGS supercontig

```
Mouse: 1       ctccggtgcctactgagctgatatcagttctcatttcacacactggctcagttcagcagg 60
               ||||||||||||||||||||||||||||||||||||||||||||||||||||||||||||
Rat:   1862421 ctccggtgcctactgagctgatatcagttctcatttcacacactggctcagttcagcagg 1862362

Mouse: 61      aacaggag 68
               ||||||||
Rat:   1862361 aacaggag 1862354
```

mmu-mir-24-2 ***Mus musculus*** **miR-24-2 precursor RNA**

>gi|34851559|ref|NW_047534.1|Rn19_2056 *Rattus norvegicus* chromosome 19 WGS supercontig

```
Mouse: 1        gcctctctccgggctccgcctcccgtgcctactgagctgaaacagttgattccagtgcac 60
                ||||||| | ||||||||||||| ||||||||||||||||||||||||||||||||||||
Rat:   10919959 gcctctccctgggctccgcctcctgtgcctactgagctgaaacagttgattccagtgcac 10919900

Mouse: 61       tggctcagttcagcaggaacaggagtccagccccc-taggagctggca 107
                ||||||||||||||||||||||||||||||||||| ||||||||||||
Rat:   10919899 tggctcagttcagcaggaacaggagtccagcccccataggagctggca 10919852
```

mmu-mir-26b ***Mus musculus*** **miR-26b precursor RNA**

>gi|34877332|ref|NW_047816.1|Rn9_2340 *Rattus norvegicus* chromosome 9 WGS supercontig

```
Mouse: 1        tgcccgggacccagttcaagtaattcaggataggttgtggtgctgaccagcctgttctcc 60
                ||||||||||||||||||||||||||||||||||||||||||||| ||||||||||||||
Rat:   17163277 tgcccgggacccagttcaagtaattcaggataggttgtggtgctggccagcctgttctcc 17163336

Mouse: 61       attacttggctcgggggccggtgcc 85
                |||||||||||||||||||||||||
Rat:   17163337 attacttggctcgggggccggtgcc 17163361
```

mmu-mir-28 ***Mus musculus*** **miR-28 precursor RNA**

>gi|34869631|ref|NW_047356.1|Rn11_1876 *Rattus norvegicus* chromosome 11 WGS supercontig

```
Mouse: 1        ggtccctaccttcaaggagctcacagtctattgagttgcctttctgattctcccactaga 60
                ||||||||||  ||||||||||||||||||||||||| | ||||||||||||||||||||
Rat:   31535044 ggtccctacccgcaaggagctcacagtctattgagttccttttctgattctcccactaga 31534985

Mouse: 61       ttgtgagctgctggagggcaggcact 86
                ||||||||| ||||||||||||||||
Rat:   31534984 ttgtgagctcctggagggcaggcact 31534959
```

mmu-mir-29a ***Mus musculus*** **miR-29a precursor RNA**

>gi|34855362|ref|NW_047689.1|Rn4_2212 *Rattus norvegicus* chromosome 4 WGS supercontig

```
Mouse: 1        accccttagaggatgactgatttcttttggtgttcagagtcaatagaattttctagcacc 60
```

```
                ||||||||||||||||||||||||||||||||||||||||||||||||||||||||||||
Rat:   28825083 accccttagaggatgactgatttcttttggtgttcagagtcaatagaattttctagcacc 28825024

Mouse: 61       atctgaaatcggttataatgattgggga 88
                ||||||||||||||||||||||||||||
Rat:   28825023 atctgaaatcggttataatgattgggga 28824996
```

mmu-mir-29c *Mus musculus* miR-29c precursor RNA

>gi|34881453|ref|NW_047404.1|Rn13_1926 *Rattus norvegicus* chromosome 13 WGS supercontig

```
Mouse: 1       atctcttacacaggctgaccgatttctcctggtgttcagagtctgtttttgtctagcacc 60
               ||||||||||||||||||||||||||||||||||||||||||||||||||||||||||||
Rat:   2146548 atctcttacacaggctgaccgatttctcctggtgttcagagtctgtttttgtctagcacc 2146607

Mouse: 61      atttgaaatcggttatgatgtaggggga 88
               ||||||||||||||||||||||||||||
Rat:   2146608 atttgaaatcggttatgatgtaggggga 2146635
```

mmu-mir-29b-1 *Mus musculus* miR-29b-1 precursor RNA

>gi|34855362|ref|NW_047689.1|Rn4_2212 *Rattus norvegicus* chromosome 4 WGS supercontig

```
Mouse: 1        aggaagctggtttcatatggtggtttagatttaaatagtgattgtctagcaccatttgaa 60
                ||||||||||||||||||||||||||||||||||||||||||||||||||||||||||||
Rat:   28825451 aggaagctggtttcatatggtggtttagatttaaatagtgattgtctagcaccatttgaa 28825392

Mouse: 61       atcagtgttct 71
                |||||||||||
Rat:   28825391 atcagtgttct 28825381
```

mmu-mir-29b-2 *Mus musculus* miR-29b-2 precursor RNA

>gi|34881453|ref|NW_047404.1|Rn13_1926 *Rattus norvegicus* chromosome 13 WGS supercontig

```
Mouse: 1       cttctggaagctggtttcacatggtggcttagatttttccatctttgtatctagcaccat 60
               ||||||||||||||||||||||||||||||||||||||||||||||||||||||||||||
Rat:   2146020 cttctggaagctggtttcacatggtggcttagatttttccatctttgtatctagcaccat 2146079

Mouse: 61      ttgaaatcagtgttttaggag 81
               |||||||||||||||||||||
Rat:   2146080 ttgaaatcagtgttttaggag 2146100
```

mmu-mir-30b *Mus musculus* miR-30b precursor RNA

>gi|34867094|ref|NW_047780.1|Rn7_2304 *Rattus norvegicus* chromosome 7 WGS supercontig

```
Mouse: 1      atgtaaacatcctacactcagctgtcatacatgcgttggctgggatgtggatgtttacgt 60
              ||||||||||||||||||||||||||||||||| ||||||||||||||||||||||||||
Rat:   418464 atgtaaacatcctacactcagctgtcatacatgagttggctgggatgtggatgtttacgt 418405
```

mmu-mir-30c-1 *Mus musculus* miR-30c-1 precursor RNA

>gi|34871316|ref|NW_047719.1|Rn5_2243 *Rattus norvegicus* chromosome 5 WGS supercontig

```
Mouse: 1       accatgttgtagtgtgtgtaaacatcctacactctcagctgtgagctcaaggtggctggg 60
               ||||||||||||||||||||||||||||||||||||||||||||||||||||||||||||
Rat:   2519905 accatgttgtagtgtgtgtaaacatcctacactctcagctgtgagctcaaggtggctggg 2519846

Mouse: 61      agagggttgtttactccttctgccatgga 89
               |||||||||||||||||||||||||||||
Rat:   2519845 agagggttgtttactccttctgccatgga 2519817
```

mmu-mir-30c-2 *Mus musculus* miR-30c-2 precursor RNA

>gi|34875263|ref|NW_047813.1|Rn9_2337 *Rattus norvegicus* chromosome 9 WGS supercontig

```
Mouse: 1        gagtgacagatattgtaaacatcctacactctcagctgtgaaaagtaagaaagctgggag 60
                |||||||||||| |||||||||||||||||||||||||||||||||||||||||||||||
```

```
Rat:   22164044 gagtgacagatactgtaaacatcctacactctcagctgtgaaaagtaagaaagctgggag 22164103

Mouse: 61       aaggctgtttactctctctgcctt 84
                ||||||||||||||||||||||||
Rat:   22164104 aaggctgtttactctctctgcctt 22164127
```

mmu-mir-92-2 ***Mus musculus*** **miR-92-2 precursor RNA**

>gi|34881658|ref|NW_048049.1|RnX_2574 *Rattus norvegicus* chromosome X WGS supercontig

```
Mouse: 1       tgcccattcatccacaggtggggattggtggcattacttgtgttagatataaagtattgc 60
               |||||||||||||||||||||||||| ||| |||||||||||||||||| ||||||||||
Rat:   2017453 tgcccattcatccacaggtggggattagtgccattacttgtgttagataaaaagtattgc 2017394

Mouse: 61      acttgtcccggcctgaggaagaaa 84
               ||||||||||||||||||||||||
Rat:   2017393 acttgtcccggcctgaggaagaaa 2017370
```

mmu-mir-93 ***Mus musculus*** **miR-93 precursor RNA**

>gi|34871632|ref|NW_047369.1|Rn12_1890 *Rattus norvegicus* chromosome 12 WGS supercontig

```
Mouse: 1       agtcatggggctccaaagtgctgttcgtgcaggtagtgtaattacctgacctactgctg 60
               |||||||||||||||||||||||||||||||||||||| | || ||||||||||||||||
Rat:   5549656 agtcatggggctccaaagtgctgttcgtgcaggtagtgca-ttgcctgacctactgctg 5549598

Mouse: 61      agctagcacttcccgagcccccaggaca 88
               ||||||||||||||||||||||||||||
Rat:   5549597 agctagcacttcccgagcccccaggaca 5549570
```

mmu-mir-99a ***Mus musculus*** **miR-99a precursor RNA**

>gi|34933964|ref|NW_047354.1|Rn11_1874 *Rattus norvegicus* chromosome 11 WGS supercontig

```
Mouse: 1        cataaacccgtagatccgatcttgtggtgaagtggaccgcgcaagctcgtttctatgggt 60
                |||||||||||||||||||||||||||||||||||||||| |||||||||||||||||||
Rat:   16397552 cataaacccgtagatccgatcttgtggtgaagtggaccgcacaagctcgtttctatgggt 16397611

Mouse: 61       ctgtg 65
                |||||
Rat:   16397612 ctgtg 16397616
```

mmu-mir-99b ***Mus musculus*** **miR-99b precursor RNA**

>gi|34854887|ref|NW_047555.1|Rn1_2077 *Rattus norvegicus* chromosome 1 WGS supercontig

```
Mouse: 1       ggcacccacccgtagaaccgaccttgcggggccttcgccgcacacaagctcgtgtctgtg 60
               ||||||||||||||||||||||||||||||||||||||||||||||||||||||||||||
Rat:   1805285 ggcacccacccgtagaaccgaccttgcggggccttcgccgcacacaagctcgtgtctgtg 1805344

Mouse: 61      ggtccgtgtc 70
               ||||||||||
Rat:   1805345 ggtccgtgtc 1805354
```

mmu-mir-103-1 ***Mus musculus*** **miR-103-1 precursor RNA**

>gi|34872015|ref|NW_047334.1|Rn10_1854 *Rattus norvegicus* chromosome 10 WGS supercontig

```
Mouse: 1       ttcttactgccctcggcttctttacagtgctgccttgttgcatatggatcaagcagcatt 60
               ||||||||||||||||||||||||||||||||||||||||||||||||||||||||||||
Rat:   7519813 ttcttactgccctcggcttctttacagtgctgccttgttgcatatggatcaagcagcatt 7519872

Mouse: 61      gtacagggctatgaaggcattgagac 86
               ||||||||||||||||||||||||||
Rat:   7519873 gtacagggctatgaaggcattgagac 7519898
```

mmu-mir-103-2 ***Mus musculus*** **miR-103-2 precursor RNA**

>gi|34859757|ref|NW_047658.1|Rn3_2180 *Rattus norvegicus* chromosome 3 WGS supercontig

```
Mouse: 1       gtcttcgtgctttcagcttctttacagtgctgccttgtagcattcaggtcaagcagcatt 60
               ||||||||||||||||||||||||||||||||||||||||||||||||||||||||||||
Rat:   9173953 gtcttcgtgctttcagcttctttacagtgctgccttgtagcattcaggtcaagcagcatt 9174012

Mouse: 61      gtacagggctatgaaagaaccaagaa 86
               ||||||||||||||||||||||||||
Rat:   9174013 gtacagggctatgaaagaaccaagaa 9174038
```

mmu-mir-124a-1 *Mus musculus* miR-124a-1 precursor RNA

>gi|34875912|ref|NW_047454.1|Rn15_1976 *Rattus norvegicus* chromosome 15 WGS supercontig

```
Mouse: 1        aggcctctctctccgtgttcacagcggaccttgatttaaatgtccatacaattaaggcac 60
                ||||||||||||||||||||||||||||||||||||||||||||||||||||||||||||
Rat:   15806503 aggcctctctctccgtgttcacagcggaccttgatttaaatgtccatacaattaaggcac 15806562

Mouse: 61       gcggtgaatgccaagaatggggctg 85
                |||||||||||||||||||||||||
Rat:   15806563 gcggtgaatgccaagaatggggctg 15806587
```

mmu-mir-124a-2 *Mus musculus* miR-124a-2 precursor RNA

>gi|34855621|ref|NW_047624.1|Rn2_2146 *Rattus norvegicus* chromosome 2 WGS supercontig

```
Mouse: 1       atcaagatcagagactctgctctccgtgttcacagcggaccttgatttaatgtcatacaa 60
               ||||||||||||||||||||||||||||||||||||||||||||||||||||||||||||
Rat:   7685115 atcaagatcagagactctgctctccgtgttcacagcggaccttgatttaatgtcatacaa 7685174

Mouse: 61      ttaaggcacgcggtgaatgccaagagcggagcctacggctgcacttgaa 109
               |||||||||||||||||||||||||||||||||||||||||||||||||
Rat:   7685175 ttaaggcacgcggtgaatgccaagagcggagcctacggctgcacttgaa 7685223
```

mmu-mir-124a-3 *Mus musculus* miR-124a-3 precursor RNA

>gi|34861141|ref|NW_047667.1|Rn3_2189 *Rattus norvegicus* chromosome 3 WGS supercontig

```
Mouse: 1      ctctgcgtgttcacagcggaccttgatttaatgtctatacaattaaggcacgcggtgaat 60
              ||||||||||||||||||||||||||||||||||||||||||||||||||||||||||||
Rat:   505484 ctctgcgtgttcacagcggaccttgatttaatgtctatacaattaaggcacgcggtgaat 505543

Mouse: 61     gccaagag 68
              ||||||||
Rat:   505544 gccaagag 505551
```

mmu-mir-125a *Mus musculus* miR-125a precursor RNA

>gi|34854887|ref|NW_047555.1|Rn1_2077 *Rattus norvegicus* chromosome 1 WGS supercontig

```
Mouse: 1       ctgggtccctgagaccctttaacctgtgaggacgtccagggtcacaggtgaggttcttgg 60
               ||||||||||||||||||||||||||||||||||||||||||||||||||||||||||||
Rat:   1805905 ctgggtccctgagaccctttaacctgtgaggacgtccagggtcacaggtgaggttcttgg 1805964

Mouse: 61      gagcctgg 68
               ||||||||
Rat:   1805965 gagcctgg 1805972
```

mmu-mir-125b-2 *Mus musculus* miR-125b-2 precursor RNA

>gi|34933964|ref|NW_047354.1|Rn11_1874 *Rattus norvegicus* chromosome 11 WGS supercontig

```
Mouse: 2        cctagtccctgagaccctaacttgtgaggtattttagtaacatcacaagtcaggttcttg 61
                |||||||||||||||||||||||||||||||||||||||||||||||||||||| |||||
Rat:   16443677 cctagtccctgagaccctaacttgtgaggtattttagtaacatcacaagtcaggctcttg 16443736

Mouse: 62       ggacctaggc 71
                ||||||||||
Rat:   16443737 ggacctaggc 16443746
```

mmu-mir-125b-1 *Mus musculus* miR-125b-1 precursor RNA

```
Mouse: 1        tgcgctcccctcagtccctgagaccctaacttgtgatgtttaccgtttaaatccacgggt 60
                ||||||||||||||||||||||||||||||||||||||||||||||||||||||||||||
Rat:   14189379 tgcgctcccctcagtccctgagaccctaacttgtgatgtttaccgtttaaatccacgggt 14189438

Mouse: 61       taggctcttgggagctg 77
                |||||||||||||||||
Rat:   14189439 taggctcttgggagctg 14189455
```

mmu-mir-127 *Mus musculus* miR-127 precursor RNA

>gi|34935858|ref|NW_047762.1|Rn6_2286 *Rattus norvegicus* chromosome 6 WGS supercontig

```
Mouse: 1        ccagcctgctgaagctcagagggctctgattcagaaagatcatcggatccgtctgagctt 60
                ||||||||||||||||||||||||||||||||||||||||||||||||||||||||||||
Rat:   29527445 ccagcctgctgaagctcagagggctctgattcagaaagatcatcggatccgtctgagctt 29527504

Mouse: 61       ggctggtcgg 70
                ||||||||||
Rat:   29527505 ggctggtcgg 29527514
```

mmu-mir-128a *Mus musculus* miR-128a precursor RNA

>gi|34933508|ref|NW_047394.1|Rn13_1916 *Rattus norvegicus* chromosome 13 WGS supercontig

```
Mouse: 1       gttggattcggggccgtagcactgtctgagaggtttacatttctcacagtgaaccggtct 60
               ||||||||||||||||||||||||||||||||||||||||||||||||||||||||||||
Rat:   1019804 gttggattcggggccgtagcactgtctgagaggtttacatttctcacagtgaaccggtct 1019863

Mouse: 61      ctttttcagc 70
               ||||||||||
Rat:   1019864 ctttttcagc 1019873
```

mmu-mir-128b *Mus musculus* miR-128b precursor RNA

>gi|34866469|ref|NW_047802.1|Rn8_2326 *Rattus norvegicus* chromosome 8 WGS supercontig

```
Mouse: 1       cagtgggaagggggggccgatgcactgtaagagagtgagtagcaggtctcacagtgaaccg 60
               ||||||||||||||||||||||||||||||||||||||||||||||||||||||||||||
Rat:   3171003 cagtgggaagggggggccgatgcactgtaagagagtgagtagcaggtctcacagtgaaccg 3170944

Mouse: 61      gtctctttccctactg 76
               ||||||||||||||||
Rat:   3170943 gtctctttccctactg 3170928
```

mmu-mir-130a *Mus musculus* miR-130a precursor RNA

>gi|34857850|ref|NW_047657.1|Rn3_2179 *Rattus norvegicus* chromosome 3 WGS supercontig

```
Mouse: 1       gagctcttttcacattgtgctactgtcta-acgtgtaccgagcagtgcaatgttaaaagg 59
               ||||||||||||||||||||||||||||| ||||||||||||||||||||||||||||||
Rat:   9350850 gagctcttttcacattgtgctactgtctacacgtgtaccgagcagtgcaatgttaaaagg 9350791

Mouse: 60      gcatc 64
               |||||
Rat:   9350790 gcatc 9350786
```

mmu-mir-132 *Mus musculus* miR-132 precursor RNA

>gi|34873416|ref|NW_047336.1|Rn10_1856 *Rattus norvegicus* chromosome 10 WGS supercontig

```
Mouse: 1       gggcaaccgtggctttcgattgttactgtgggaaccggaggtaacagtctacagccatgg 60
               ||||||||||||||||||||||||||||||||||||||||||||||||||||||||||||
Rat:   3479949 gggcaaccgtggctttcgattgttactgtgggaaccggaggtaacagtctacagccatgg 3480008

Mouse: 61      tcgccc 66
               ||||||
Rat:   3480009 tcgccc 3480014
```

mmu-mir-136 *Mus musculus* miR-136 precursor RNA

```
>gi|34935858|ref|NW_047762.1|Rn6_2286 Rattus norvegicus chromosome 6 WGS
                supercontig

Mouse: 1        gaggactccatttgttttgatgatggattcttaagctccatcatcgtctcaaatgagtct 60
                ||||||||||||||||||||||||||||||||||||||||||||||||||||||||||||
Rat:   29530106 gaggactccatttgttttgatgatggattcttaagctccatcatcgtctcaaatgagtct 29530165

Mouse: 61       tc 62
                ||
Rat:   29530166 tc 29530167
```

mmu-mir-138-1 *Mus musculus* miR-138-1 precursor RNA

```
>gi|34866724|ref|NW_047804.1|Rn8_2328 Rattus norvegicus chromosome 8 WGS
              supercontig

Mouse: 1      ctctagcatggtgttgtgggacagctggtgttgtgaatcaggccgttgccaatcagagaa 60
              |||| |||||||||||||||||||||||||||||||||||||||||||||||||||||||
Rat:   156597 ctctggcatggtgttgtgggacagctggtgttgtgaatcaggccgttgccaatcagagaa 156656

Mouse: 61     cggctacttcacaacaccagggccacactgcactgca 97
              |||||||||||||||||||||| | ||||||||||||
Rat:   156657 cggctacttcacaacaccagggtctcactgcactgca 156693
```

mmu-mir-138-2 *Mus musculus* miR-138-2 precursor RNA

```
>gi|34851309|ref|NW_047531.1|Rn19_2053 Rattus norvegicus chromosome 19 WGS
                supercontig

Mouse: 1        cagctggtgttgtgaatcaggccgacgagcagcgcatcctcttacccggctatttcacga 60
                ||||||||||||||||||||||||||||||| ||||||||||||||||||||||||||||
Rat:   11119560 cagctggtgttgtgaatcaggccgacgagcaacgcatcctcttacccggctatttcacga 11119501

Mouse: 61       caccagggttg 71
                |||||||||||
Rat:   11119500 caccagggttg 11119490
```

mmu-mir-139 *Mus musculus* miR-139 precursor RNA

```
>gi|34860310|ref|NW_047562.1|Rn1_2084 Rattus norvegicus chromosome 1 WGS
               supercontig

Mouse: 1       gtgtattctacagtgcacgtgtctccagtgtggctcggaggctggagacgcggccctgtt 60
               ||||||||||||||||||||||||||||||||||||||||||||||||||||||||||||
Rat:   1586714 gtgtattctacagtgcacgtgtctccagtgtggctcggaggctggagacgcggccctgtt 1586773

Mouse: 61      ggagtaac 68
               ||||||||
Rat:   1586774 ggagtaac 1586781
```

mmu-mir-142 *Mus musculus* miR-142 precursor RNA

```
>gi|34873416|ref|NW_047336.1|Rn10_1856 Rattus norvegicus chromosome 10 WGS
                supercontig

Mouse: 1        acccataaagtagaaagcactactaacagcactggagggtgtagtgtttcctactttatg 60
                ||||||||||||||||||||||||||||||||||||||||||||||||||||||||||||
Rat:   17049244 acccataaagtagaaagcactactaacagcactggagggtgtagtgtttcctactttatg 17049303

Mouse: 61       gatg 64
                ||||
Rat:   17049304 gatg 17049307
```

mmu-mir-145 *Mus musculus* miR-145 precursor RNA

```
>gi|34932227|ref|NW_047514.1|Rn18_2036 Rattus norvegicus chromosome 18 WGS
               supercontig

Mouse: 1       ctcacggtccagttttcccaggaatcccttggatgctaagatggggattcctggaaatac 60
               ||||||||||||||||||||||||||||||||||||||||||||||||||||||||||||
Rat:   3665639 ctcacggtccagttttcccaggaatcccttggatgctaagatggggattcctggaaatac 3665580

Mouse: 61      tgttcttgag 70
               ||||||||||
Rat:   3665579 tgttcttgag 3665570
```

mmu-mir-146 ***Mus musculus*** **miR-146 precursor RNA**

>gi|34872015|ref|NW_047334.1|Rn10_1854 *Rattus norvegicus* chromosome 10 WGS supercontig

```
Mouse: 1        agctctgagaactgaattccatgggttatatcaatgtcagacctgtgaaattcagttctt 60
                |||||||||||||||||||||||||||||| |||||||||||||||||| ||||||||||
Rat:   15301237 agctctgagaactgaattccatgggttatagcaatgtcagacctgtgaagttcagttctt 15301178

Mouse: 61       cagct 65
                 ||||
Rat:   15301177 tagct 15301173
```

mmu-mir-150 ***Mus musculus*** **miR-150 precursor RNA**

```
Mouse: 1       ccctgtctcccaacccttgtaccagtgctgtgcctcagaccctggtacaggcctgggggga 60
               ||||||||||||||||||||||||||||||||||||||||||||||||||||||||||||
Rat:   4552248 ccctgtctcccaacccttgtaccagtgctgtgcctcagaccctggtacaggcctgggggga 4552307

Mouse: 61      taggg 65
                ||||
Rat:   4552308 caggg 4552312
```

mmu-mir-154 ***Mus musculus*** **miR-154 precursor RNA**

```
Mouse: 1        gaagataggttatccgtgttgccttcgctttattcgtgacgaatcatacacggttgacct 60
                ||||||||||||||||||||||||||||||||||||||||||||||||||||||||||||
Rat:   29762799 gaagataggttatccgtgttgccttcgctttattcgtgacgaatcatacacggttgacct 29762858

Mouse: 61       attttt 66
                ||||||
Rat:   29762859 attttt 29762864
```

mmu-mir-185 ***Mus musculus*** **miR-185 precursor RNA**

>gi|34869997|ref|NW_047358.1|Rn11_1878 *Rattus norvegicus* chromosome 11 WGS supercontig

```
Mouse: 1       agggattggagagaaaggcagttcctgatggtcccctcccaggggctggctttcctctgg 60
               ||||||||||||||||||||||||||||||||||||||||||||||||||||||||||||
Rat:   2687772 agggattggagagaaaggcagttcctgatggtcccctcccaggggctggctttcctctgg 2687831

Mouse: 61      tcctt 65
               |||||
Rat:   2687832 tcctt 2687836
```

mmu-mir-191 ***Mus musculus*** **miR-191 precursor RNA**

>gi|34866469|ref|NW_047802.1|Rn8_2326 *Rattus norvegicus* chromosome 8 WGS supercontig

```
Mouse: 1     agcgggcaacggaatcccaaaagcagctgttgtctccagagcattccagctgcacttgga 60
             ||||||||||||||||||||||||||||||||||||||||||||||||||||||||||||
Rat:   58055 agcgggcaacggaatcccaaaagcagctgttgtctccagagcattccagctgcacttgga 58114

Mouse: 61    tttcgttccctgct 74
             ||||||||||||||
Rat:   58115 tttcgttccctgct 58128
```

mmu-mir-213 ***Mus musculus*** **miR-213 precursor RNA**

>gi|34880444|ref|NW_047396.1|Rn13_1918 *Rattus norvegicus* chromosome 13 WGS supercontig

```
Mouse: 1       ggttgcttcagtgaacattcaacgctgtcggtgagtttggaattcaaataaaaaccatcg 60
               ||||||||||||||||||||||||||||||||||||||||||||||||||||||||||||
Rat:   1976067 ggttgcttcagtgaacattcaacgctgtcggtgagtttggaattcaaataaaaaccatcg 1976126

Mouse: 61      accgttgattgtaccctatagctaacc 87
               |||||||||||||||||||||||||||
Rat:   1976127 accgttgattgtaccctatagctaacc 1976153
```

mmu-mir-300 ***Mus musculus*** **miR-300 precursor RNA**

>gi|34935858|ref|NW_047762.1|Rn6_2286 *Rattus norvegicus* chromosome 6 WGS supercontig

```
Mouse: 1        gctacttgaagagaggttatcctttgtgtgtttgctttacgcgaaatgaatatgcaaggg 60
                ||||||||||||||||||||||||||||||||||||||||||||||||||||||||||||
Rat:   29748356 gctacttgaagagaggttatcctttgtgtgtttgctttacgcgaaatgaatatgcaaggg 29748415

Mouse: 61       caagctctcttcgaggagc 79
                |||||||||||||||||||
Rat:   29748416 caagctctcttcgaggagc 29748434
```

<table>
<tr><th>Rat microRNA</th><th>Predicted folding Energy(kcal/mole)</th><th>Predicted stem loop precursor</th></tr>
<tr><td>rno-let-7a-2</td><td>dG = -38.9</td><td><pre>
10   20   30   40
--| UGC   CU   G   U   UAGAGUUACA
  GCA   UCCCAGG GAG UAG AGGUUGUAUAGUU   \
  CGU   AGGGUUC UUC AUC UCCGACAUGUCAA   A
CA^ UC-   CU   G   C   UAGAGGGAAC
   90   80   70   60   50
</pre></td></tr>
<tr><td>rno-let-7b</td><td>dG = -44.0</td><td><pre>
---| U   - -----   CA   A
   GGG GAGGUAGUAGGUUGUGUGGUU UC   AGGG GUG U
   CCC UUCCGUCAUCCAACAUAUCAA AG   UCCC CGC G
AGU^ -   U AAGCC   --   U
</pre></td></tr>
<tr><td>rno-mir-7-1</td><td>dG = -46.8</td><td><pre>
U--| U U   A U   A   A   U   --   A
   UGGA GU GGCCU GU CUGUGUGG AGACU GUGAUUU GUUGUU UUUAG U
   AUCU CA CCGGA CA GGUAUACC UCUGA CACUAAA CAACAG GAAUC A
GAC^   C -   - C   G   -   -   CA   A
</pre></td></tr>
<tr><td>rno-mir-7-2</td><td>dG = -29.1</td><td><pre>
---| C   C   A   AGU   U   UG C
   CCAGC CUGU UGG AGACU   GAUUU GUUGUUG U U
   GGUUG GGUA ACC UCUGA   CUGAA CAACAAC G G
ACU^   U   C   G   CC-   -   CU U
</pre></td></tr>
<tr><td>rno-mir-16-2</td><td>dG = -36.9</td><td><pre>
----| UC   CU   UA   C AG   AAU
    CUUGU CGCU AGCAGCACG AAUAUUGG GU UGA   A
    GGACA GUGA UCGUCGUGU UUAUAACC CA AUU   A
AUAG^   GU   UU   UA   A A-   AUA
</pre></td></tr>
<tr><td>rno-mir-24-2</td><td>dG = -53.5</td><td><pre>
-| U-   CU---   CCGC   G   A   AA   UG U
 GCC CUCC   GGGCU   CUCCUGU CCU CUGAGCUGA CAGU AU C
 CGG GAGG   CCCGA   GAGGACA GGA GACUUGACU GUCA UG C
A^ UC   AUACC   CCU-   A   C   CG   CG A
</pre></td></tr>
<tr><td>rno-mir-26b</td><td>dG = -42.6</td><td><pre>
U| -   GA   -   U   UC   UG   G
 GC CCGG CCC AGU CAAGUAAU AGGAUAGGU UGGU C
 CG GGCC GGG UCG GUUCAUUA UCUUGUCCG ACCG U
C^ U   GG   C   -   CC   --   G
</pre></td></tr>
<tr><td>rno-mir-28</td><td>dG = -41.2</td><td><pre>
| C   A   GCA   UU--   U- CUU
GGU CCU CCC   AGGAGCUCACAGUCUA   GAG UC   \
UCA GGA GGG   UCCUCGAGUGUUAGAU   CUC AG   U
^ C   C   AGG   CACC   UU UCU
</pre></td></tr>
<tr><td>rno-mir-30b</td><td>dG = -28.1</td><td><pre>
| U   -   --   U
AUGUAAACAUCC ACA CUCAGCUG UCA A
UGCAUUUGUAGG UGU GGGUCGGU AGU C
^   -   A   UG   A
</pre></td></tr>
<tr><td>rno-mir-30c-2</td><td>dG = -28.0</td><td><pre>
GAGUGA| UACU   U   ACA   GUGAAA
      CAGA   GUAAACA CCU   CUCUCAGCU   A
      GUCU   CAUUUGU GGA   GAGGGUCGA   G
UUCC--^   CUCU   C   A--   AAGAAU
</pre></td></tr>
<tr><td>rno-mir-92-2</td><td>dG = -36.5</td><td><pre>
UGCCCA| A   A   G   U   CAU   GUGUU
      UUC UCC CAGGU GGGAU AGUGC   UACUU   A
      AAG AGG GUCCG CCCUG UCACG   AUGAA   G
A-----^ A   A   G   U   UU-   AAAUA
</pre></td></tr>
<tr><td>rno-mir-93</td><td>dG = -48.2</td><td><pre>
A| A   CA-   -   U   G   UG UU
 GUC UGGGGGCUC   AAGUGCU GUUCG GCAG UAG CA \
 CAG ACCCCCGAG   UUCACGA CGAGU CGUC AUC GU G
A^ G   CCC   U   -   -   CA CC
</pre></td></tr>
</table>

rno-mir-99a	dG = -29.5	| A UC U G AAG<br>CAUA ACCCGUAGA CGA CUUGUG UG U<br>GUGU UGGGUAUCU GCU GAACAC GC G<br>^ C UU C - CAG
rno-mir-125b-2	dG = -33.3	-| UC UG C A GG- U<br>CCUAG CC AGA CCU ACUUGUGA UAU U<br>GGAUC GG UCU GGA UGAACACU AUG U<br>C^ CA GU C C ACA A
rno-mir-130a	dG = -24.7	GA-| C UG A GUC A<br>GCUCUUUU ACAUUG CU CU UAC C<br>CGGGAAAA UGUAAC GA GA AUG G<br>CUA^ U GU C GCC U
rno-mir-138-1	dG = -50.6	CUCUG| UG U AG UCA GCCAA<br>GCA GUGU GUGGGAC CUGGUGUUGUGAA GGCCGUU \\<br>CGU CACG CACUCUG GACCACAACACUU UCGGCAA U<br>A----^ -- U G- CA- GAGAC
rno-mir-138-2	dG = -29.1	CAG--| UCA AC--- CAAC<br>CUGGUGUUGUGAA GGCCG GAG \\<br>GACCACAGCACUU UCGGC CUC G<br>GUUGG^ UA- CCAUU CUAC
rno-mir-146	dG = -29.3	| CU C -- G<br>AGCU GAGAACUGAAUU CAUGGGUU AUA C<br>UCGA UUCUUGACUUGA GUGUCCAG UGU A<br>^ U- A AC A
