# Supplementary material for: Microarray analysis of microRNA expression in the developing mammalian brain
Source: Genome Biol. 2004 Aug 31;5(9):R68. doi: 10.1186/gb-2004-5-9-r68 (PMC522875; doi:10.1186/gb-2004-5-9-r68)
Supplement: Additional data file 2 — A file containing precursor sequences and secondary structure predictions for the novel microRNA miR-421 [file gb-2004-5-9-r68-s2.pdf]

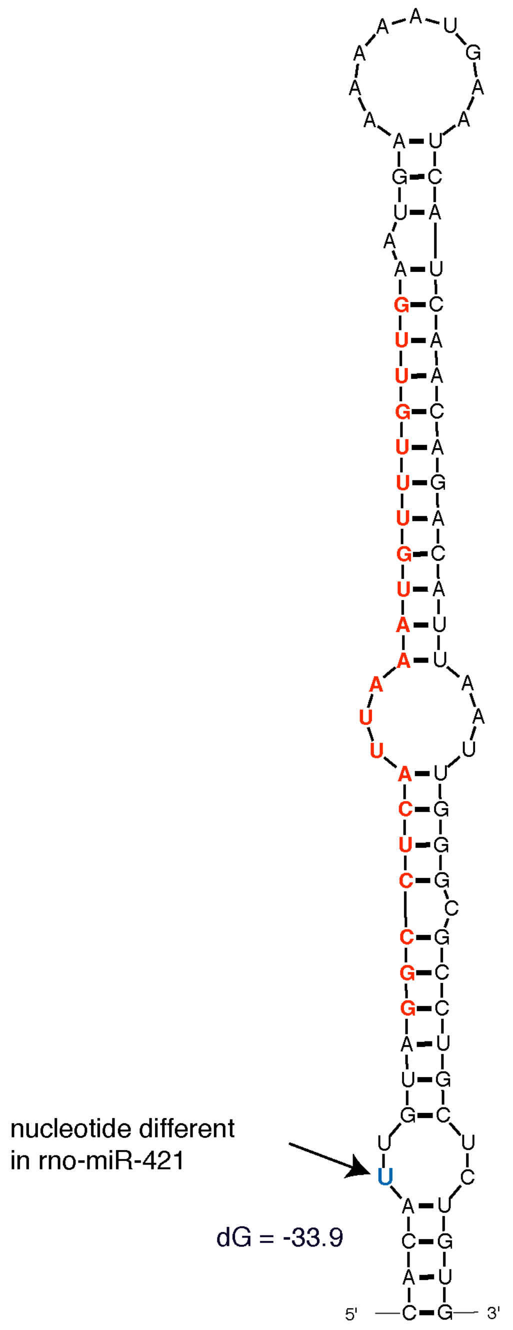

mmu-miR-421, hsa-miR-421

Figure AF2b

Figure AF2a - Alignment of precursor sequences for microRNA mir-421

ref|NW_048043.1|RnX_2568 *Rattus norvegicus* chromosome X WGS supercontig

R. norvegicus	NW_048043.1	3077508	CACACTGTA**GGCCTCATTAAATGTTTGTTG**AATGAAAAAATGAATCATCAACAGACATTAATTGGGCGCCTGCTCTGTG	3077429
H. sapiens	AC004386.1	5060	CACATTGTA**GGCCTCATTAAATGTTTGTTG**AATGAAAAAATGAATCATCAACAGACATTAATTGGGCGCCTGCTCTGTG	4982
M. musculus	AL683845.15	39424	CACATTGTA**GGCCTCATTAAATGTTTGTTG**AATGAAAAAATGAATCATCAACAGACATTAATTGGGCGCCTGCTCTGTG	39346
			*	
