# Supplementary figures and images for: Microarray analysis of microRNA expression in the developing mammalian brain
Source: Genome Biol. 2004 Aug 31;5(9):R68. doi: 10.1186/gb-2004-5-9-r68 (PMC522875; doi:10.1186/gb-2004-5-9-r68)

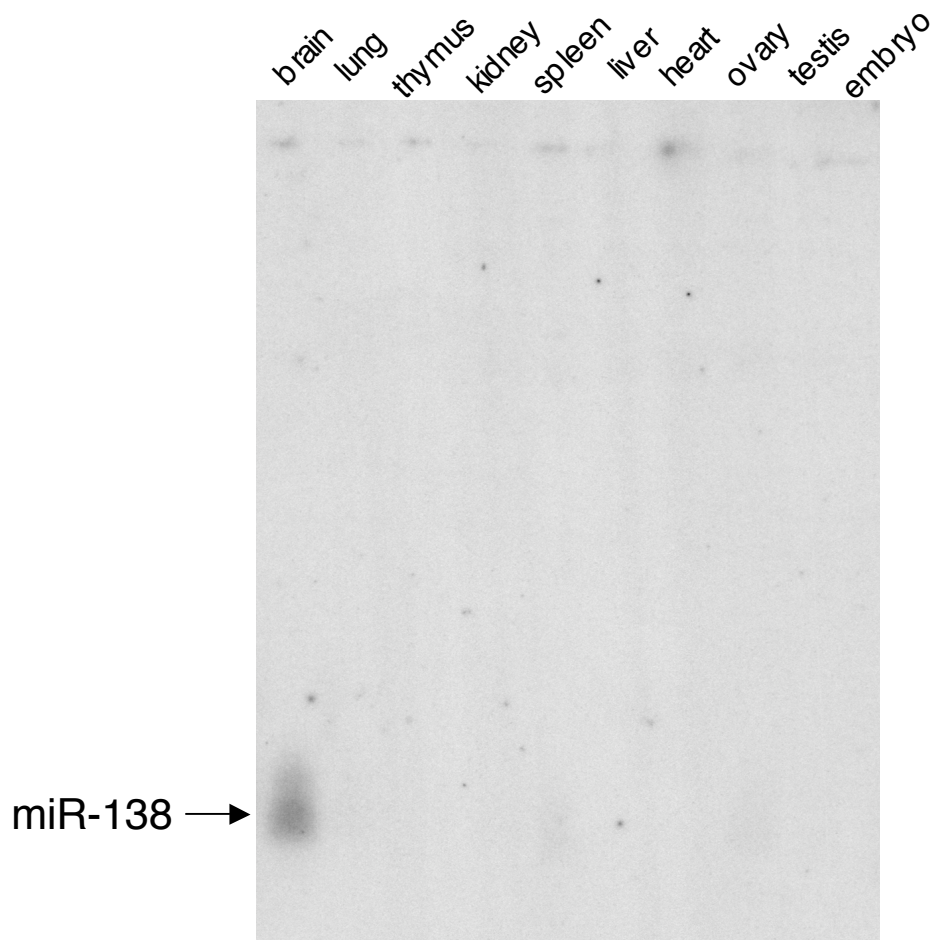

Figure AF4

Supplement: Additional data file 4 — A file showing rno-miR-138 brain specific expression [file gb-2004-5-9-r68-s4.pdf]
