# Supplementary material for: Microarray analysis of microRNA expression in the developing mammalian brain
Source: Genome Biol. 2004 Aug 31;5(9):R68. doi: 10.1186/gb-2004-5-9-r68 (PMC522875; doi:10.1186/gb-2004-5-9-r68)
Supplement: Additional data file 3 — A file with details of other small RNAs cloned from rat and monkey brains [file gb-2004-5-9-r68-s3.pdf]

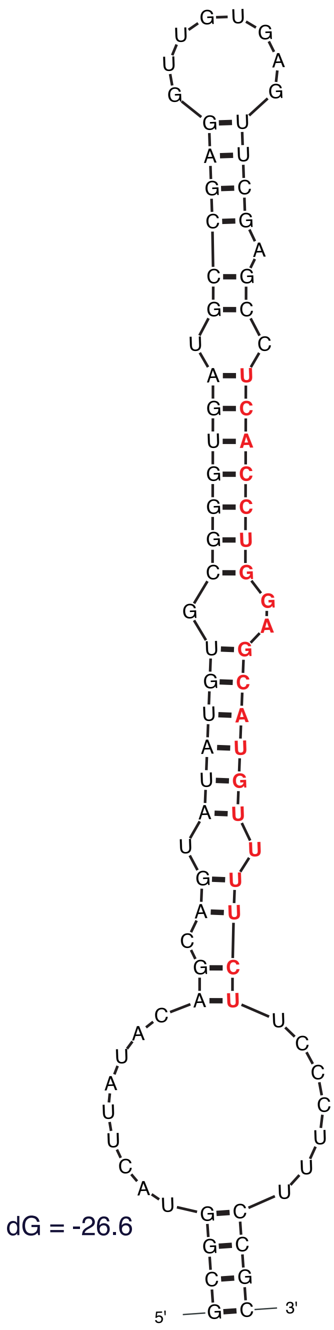

smallRNA-1

Figure AF3
