# Supplementary material for: Microarray analysis of microRNA expression in the developing mammalian brain
Source: Genome Biol. 2004 Aug 31;5(9):R68. doi: 10.1186/gb-2004-5-9-r68 (PMC522875; doi:10.1186/gb-2004-5-9-r68)
Supplement: Additional data file 5 — A file with details of microarray design and data analysis [file gb-2004-5-9-r68-s5.pdf]

Additional file 5 – Microarray design and data analysis (AF 5)

Samples/Hybridizations

Samples were processed as shown in Figure AF5a. For each sample, two independent ligations were performed. The products of the two ligations were split, and two independent reverse transcription/amplifications/hybridizations were performed. Thus, for each sample data were collected from four independent array hybridizations.

Arrays/Normalization

Glass slides were arrayed using quadruplicate spots for each of the 138 microRNA probes, 19 small RNA probes and control probes (Figure AF5a and Additional file 6). A sample microarray scan is shown in Figure AF5b. The microarray consists of 16 squares, of eight by seven spots each. Quadruplicates can be identified as rows of four spots (every other spot) in the individual squares. A TIFF file of the scanned array is used for subsequent array analysis (Digital Genome System Suite, MolecularWare). Normalized spot intensities were used for all data analysis. For inter-array comparisons, all data were scaled based on total array intensities (scaling factors ranged from 0.4 to 2.6), and data for each sample and each gene were averaged and the standard error of the mean (SEM) was calculated. Total array intensity was calculated as the sum of the normalized spot intensity for all spots in the microarray. Analysis of variance (ANOVA) performed out using Spotfire DecisionSite (Spotfire). Hierarchical clustering was performed using CLUSTER 3.0/TreeView software. For CLUSTER 3.0/TreeView output see Figure

AF5c: Profile of microRNA expression in the developing mouse brain. Colors indicate relative signal intensities. The microRNA expression profile was sorted using a hierarchical clustering method (see above). Only data from 66 probes that changed at least two-fold over the developmental time course (ANOVA, $P < 0.001$) are shown. The data used for the analysis are available as Additional file 6.

Control probes

Control probes were either negative controls or mismatch controls. The negative controls were either a synthetic $(GCAT)_n$ oligonucleotide (EAM1100) or sequences derived from mouse mRNA sequences (EAM1101-1104):

oligo ID	oligo sequence	mRNA
EAM1100	GCATGCATGCATGCATGCATG	Synthetic
EAM1101	GTGGTAGCGCAGTGCGTAGAA	beta-tubulin
EAM1102	GGTGATGCCCTGAATGTTGTC	histone H4
EAM1103	TGTCATGGATGACCTTGGCCA	glyceraldehyde dehydrogenase
EAM1104	CTTTTGACATTGAAGGGAGCT	laminin alpha 4

The mismatch controls were probes with two mismatches to a specific microRNA probe. Two central Cs were replaced by two Gs; if this change was not possible, one C was replaced with a G and one T with an A. Averaged, normalized spot intensities for these negative controls ranged from –0.8 to 1.2, as compared with 90 to 14,250 for microRNA

probes that we scored as signals. Negative values were the consequence of the subtraction of the local background signal surrounding each spot on the arrays.

Expression levels and microarray correlations

We employed two methods to distinguish signal versus noise. First, we used correlation analysis among the four hybridizations for a given time point to assess reproducibility. Second, we used a set of negative control probes (see above) to measure noise. As an example, Figure AF5d shows the correlations (scatter plots) among the four hybridizations for time point E12.5. For each graph the axes show averaged mean spot intensities for all probes from a given data set, as indicated. Arbitrarily, we chose a cut-off of 90 for our analysis. Expressed relative to background values, a microRNA was identified as being present only if the signal was at least 75-fold over that of the negative controls for at least one timepoint. Figure AF5e shows the correlations (scatter plots) of the data for E12.5 with the data from each other timepoint (each averaged over four hybridizations). As expected, the correlation between E12.5 and E17.5 is highest, and the correlation decreases with samples from more distant developmental stages.

Specificity index

To assess probe specificity, we compared the signal from oligonucleotides (probes) complementary to microRNAs (matched probe) and oligonucleotides with mismatches (mismatched probe, see above). Mismatched oligonucleotides were printed for the first 24 probes (EAM101, EAM103, … EAM147) and were named EAM102, EAM104, … EAM148. Mismatched oligonucleotides were spotted as nearest neighbors to microRNA

oligonucleotides. To calculate the specificity index (Figure 2*a*) we used datasets from two samples of each of the five time points from this study (5 time points × 2 independent samples = 10 hybridizations total). Calculations were based on cumulative signals from all experiments. EAM141, EAM143, EAM145 and EAM147 are *let*-7 family members and have very similar sequences. EAM117, EAM119 and EAM107 and EAM109 are also closely related. Therefore, there might be cross-reactivity within each of these groups. The matched/mismatched probe pair EAM135/EAM136 was excluded from Figure 2*a* as EAM136 did not give a signal above background at any of the five time points.

Summary of features on the microarray (probe set)

Mouse microRNAs (The miRNA Registry 3.2)	129
Other mammalian microRNAs (The miRNA Registry 3.2, rat and human)	9
Other unique small RNAs	18
Total	156

Of the 156 unique small RNA probes, we found that 116 (74 %) showed robust signals at at least one of the five timepoints. Of these, 83 changed significantly (ANOVA, $P < 0.001$) and 66 more than two-fold.

RNA signal quantification

The following three artifical RNAs were synthesized (Dharmacon): syn1 5'-P-CAGUCAGUCAGUCAGUCAGUCAG-3', syn2 5'-P-GACCUCCAUGUAAACGUACAA and syn3 5'-P-UUGCAGAUAACUGGUACAAG-3'. Sequences were chosen at random and were generated based on the following criteria: approximately 50% GC content, no self-annealing, no significant sequence similarity to any known mammalian microRNA and no significant sequence complementarity to any probe on the microarray. A microarray similar to the one described in this manuscript was printed with the addition of probes that are the reverse complement of these control RNAs. To quantify microRNA microarray signals the artifical RNAs were mixed with 50 μg of total RNA (an equal mix of total RNA from all five timepoints used in this study). RNA was labeled as described. The total amounts of each RNA used were 0, 0.025, 0.1, 0.375, 0.75, 2.5, 5 and 10 fmoles. Without the addition of these RNAs the normalized signal intensities were indistinguishable from background signals, which was 9 ± 6.5 (S.D.).

Analysis of base-bias of RNA ligation

	Terminal base composition of microRNAs that can be detected by the array		microRNAs expressed robustly (as defined above)	
Nucleotide	5' end	3' end	5' end	3'end
U	57	92	77% (44/57)	77% (71/92)
G	37	12	73% (27/37)	33% (4/12)
A	31	21	61% (19/31)	71% (15/21)
C	31	31	81% (25/31)	81% (25/31)

We calculated the terminal base composition of microRNAs detected robustly or not detected in our array and found that there does not appear to be a significant bias in base composition. This observation suggests that the ligation efficiency does not vary strongly depending on the identity of the 5’ or 3’ nucleotide and, as a consequence, that the method accurately reflects the microRNA content of the samples analyzed.

For the microRNAs that can be detected on the microarray, sequences with a U at the 5’ end are slightly overrepresented (57 out of 157) while sequences with U at the 3’ end are overrepresented (97 out of 157). Sequences with a G at the 3’ are underrepresented (12 out of 157). When these biases are taken into account, the percentage of microRNAs that can be detected over background ranges between 61 and 81 for the four bases at the 5’ end. While only 33% of microRNAs with G at the 3’ end can be detected, the significance of this apparently low number is difficult to determine. First, this fraction is based only on a small number of oligonucleotides in the array (12). Second, we and others have observed that the 3’ end of cloned microRNAs is variable. For example, in Table 1 we show that for microRNAs that we cloned more than once 14 of the 30 rat microRNAs and 14 of the 26 monkey microRNAs had variable 3’ ends. Thus, it is difficult to assess the significance of a correlation between base composition at the 3’end and detection of expression.

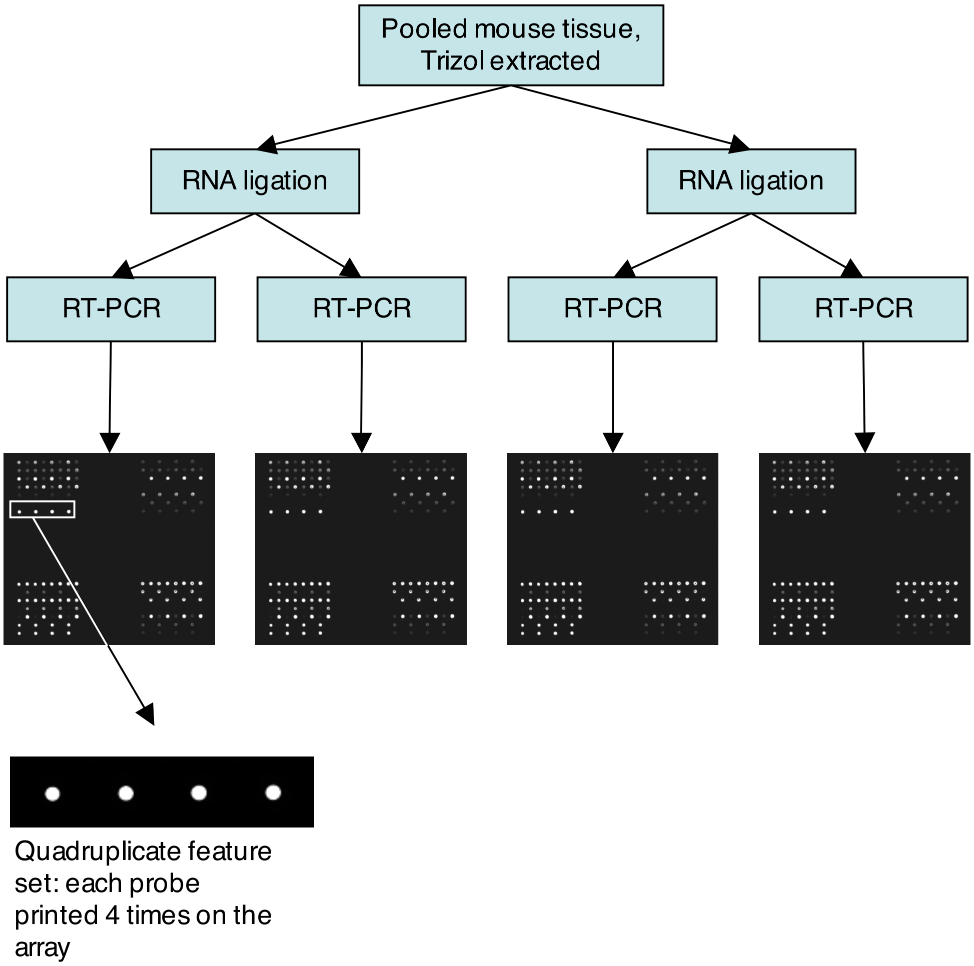

Pooled mouse tissue,
Trizol extracted
RNA ligation
RNA ligation
RT-PCR
RT-PCR
RT-PCR
RT-PCR
Quadruplicate feature
set: each probe
printed 4 times on the
array

Figure AF5a

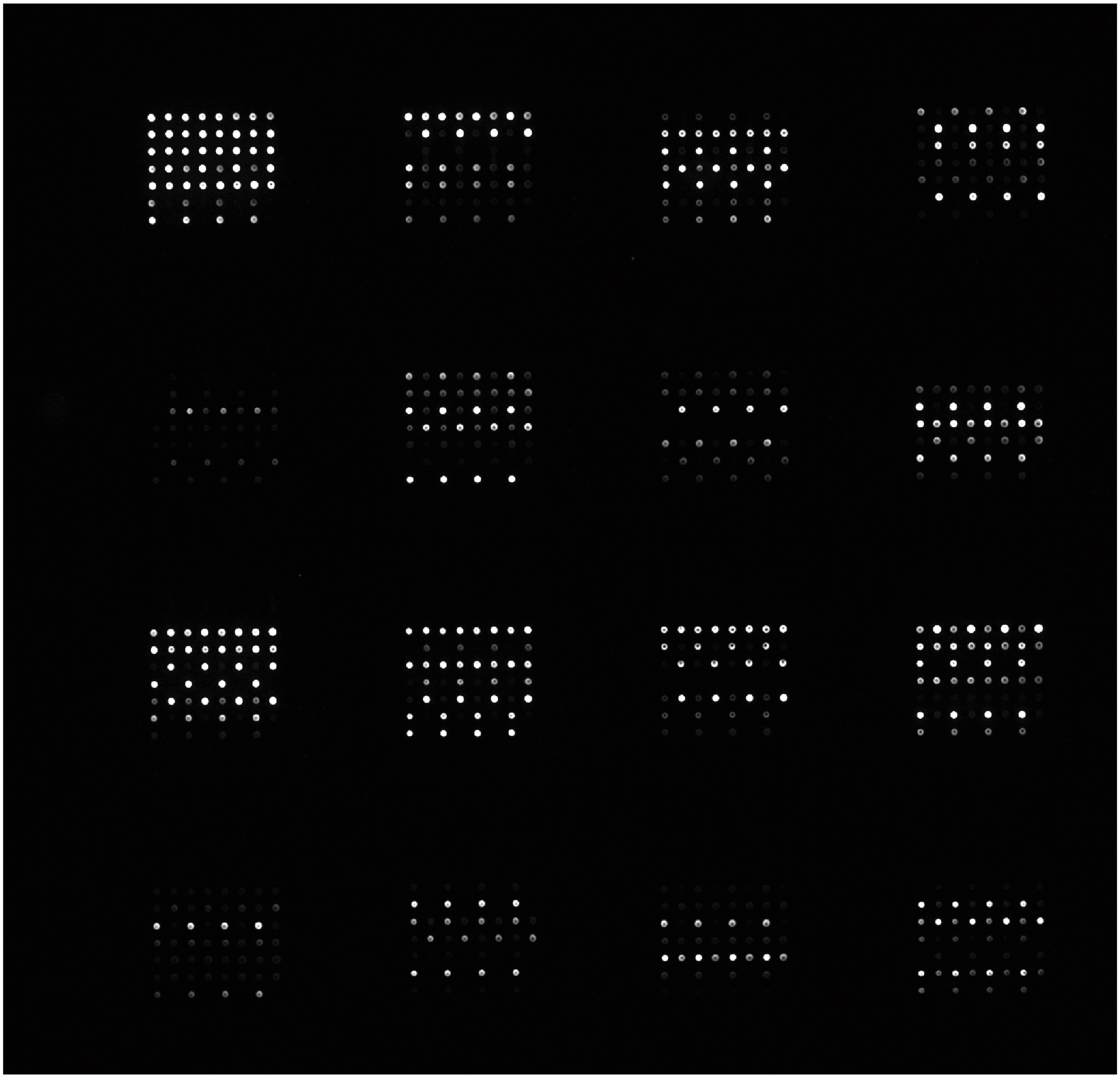

Figure AF5b. Hybridization Sample

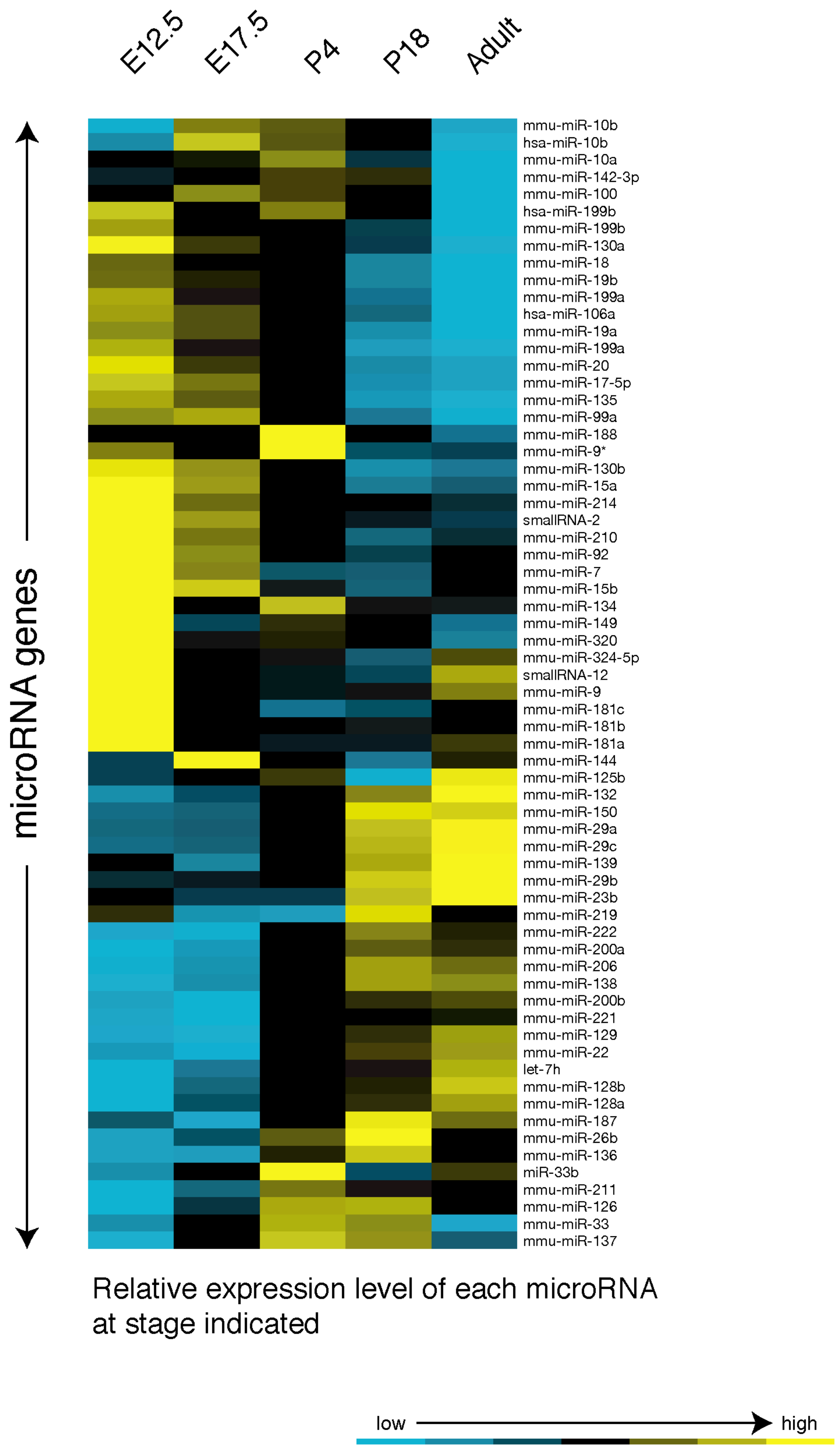

Figure AF5c. Microarray data analysis

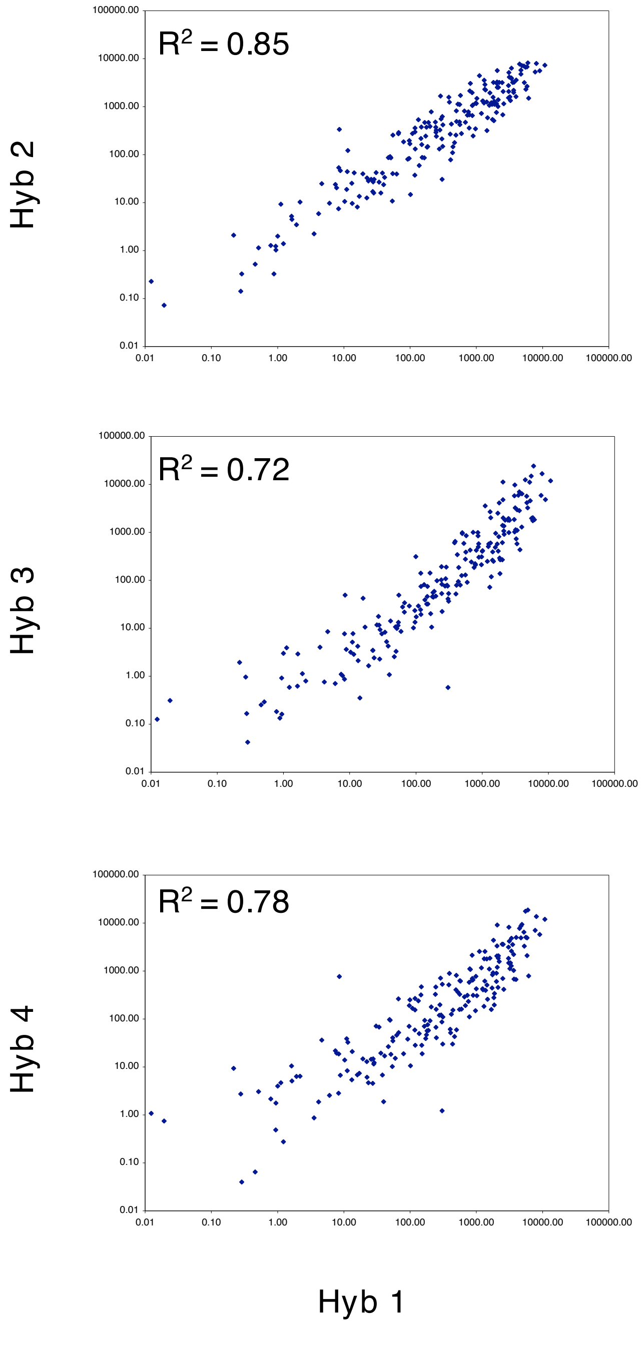

Figure AF5d

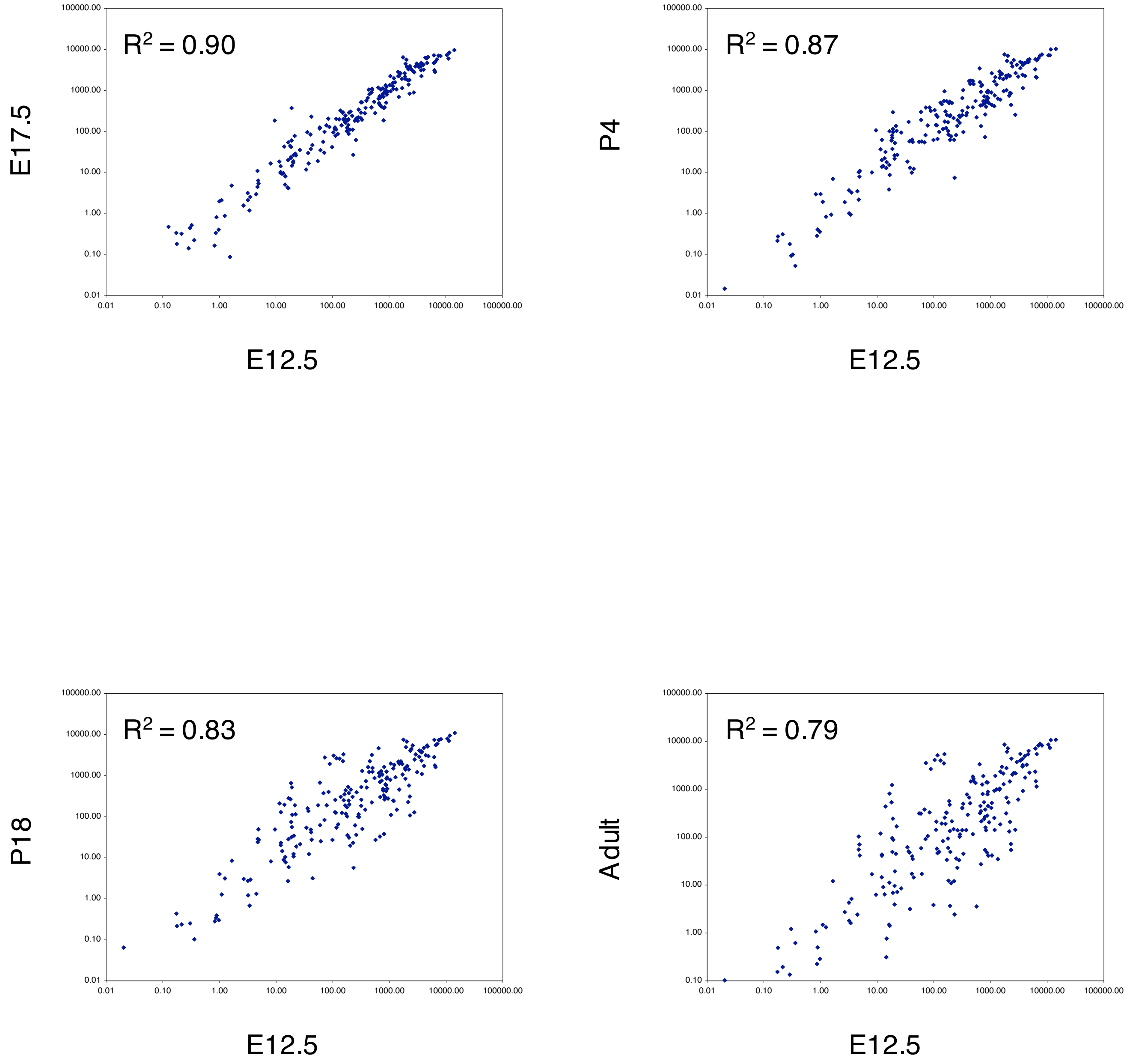
R2 = 0.90
E17.5
E12.5
R2 = 0.87
P4
E12.5
R2 = 0.83
P18
E12.5
R2 = 0.79
Adult
E12.5
100000.00
10000.00
1000.00
100.00
10.00
1.00
0.10
0.01

Figure AF5e
